# Supplementary material for: Delayed increase in stone tool cutting-edge productivity at the Middle-Upper Paleolithic transition in southern Jordan
Source: Nat Commun. 2024 Feb 7;15:610. doi: 10.1038/s41467-024-44798-y (PMC10850154; doi:10.1038/s41467-024-44798-y)
Supplement: Supplementary file 1 — Supplementary Information [file 41467_2024_44798_MOESM1_ESM.pdf]

SUPPLEMENTARY INFORMATION

Delayed increase in stone tool cutting-edge productivity at the Middle-Upper Paleolithic transition in southern Jordan

Seiji Kadowaki [1,*], Joe Yuichiro Wakano [2], Toru Tamura [3], Ayami Watanabe [1], Masato Hirose [4], Eiki Suga [5], Kazuhiro Tsukada [1], Oday Tarawneh [6] and Sate Massadeh [7]

[1] Nagoya University Museum, Nagoya University, Furo-cho, Chikusa-ku, Nagoya 464-8601, Japan

[2] School of Interdisciplinary Mathematical Sciences, Meiji University, Nakano 4-21-1, Nakano-ku, Tokyo 164-8525, Japan

[3] Geological Survey of Japan, AIST, Central 7, 1-1-1 Higashi, Tsukuba, Ibaraki 305-8567, Japan

[4] Laboratory of Archaeology, Kiso Regional Union, Nagano 399-6101, Japan

[5] Graduate School of Environmental Studies, Nagoya University, Furo-cho, Chikusa-ku, Nagoya 464-8601, Japan

[6] Department of Antiquities, Third Circle, Jabal Amman, Amman, Jordan

[7] Ministry of Tourism and Antiquities, Third Circle, Jabal Amman, Amman, Jordan

* Corresponding author

E-mail address: kadowaki@num.nagoya-u.ac.jp (S. Kadowaki).

Supplementary Note 1. Recent recognitions about the production rate of stone tool cutting-edges

Supplementary Note 2. Background of the Initial Upper Paleolithic

Supplementary Discussion. Changes in cutting-edge production rates from the Late Middle Paleolithic to the Initial Upper Paleolithic

Supplementary Fig. 1. Overview, plan map, and stratigraphic section of Tor Faraj

Supplementary Fig. 2. Overview, plan map, and stratigraphic section of Tor Sabiha

Supplementary Fig. 3. Overview, plan map, and stratigraphic section of Wadi Aghar

Supplementary Fig. 4. Overview, plan map, and stratigraphic section of Tor Fawaz

Supplementary Fig. 5. Overview, plan map, and stratigraphic section of Tor Hamar

Supplementary Fig. 6. Some typical samples of flaked stone artifacts analyzed in this study

Supplementary Fig. 7. Summarized methods for quantifying the productivity of stone tool cutting-edges

Supplementary Fig. 8. Scatterplots between the edge length per mass (mm/g) of complete lithic specimens and their mass

Supplementary Fig. 9. Scatterplots between the edge length per mass (mm/g) of complete lithic specimens and their thickness

Supplementary Fig. 10. Scatterplots between the edge length per mass (mm/g) of complete lithic specimens and the platform area

Supplementary Fig. 11. Scatterplots between the edge length per mass (mm/g) of complete lithic specimens and their length

Supplementary Fig. 12. Scatterplots between the edge length per mass (mm/g) of complete lithic specimens and the ratio of length to width (i.e., elongatedness)

Supplementary Fig. 13. Scatterplots between the edge length per mass (mm/g) of complete lithic specimens and the ratio of width to thickness (i.e., flatness)

Supplementary Fig. 14. Diachronic changes in width of complete blanks in the eight assemblages from LMP to Epi

Supplementary Fig. 15. Diachronic changes in thickness of complete blanks in the eight assemblages from LMP to Epi

Supplementary Fig. 16. Diachronic changes in the platform area of complete blanks in the eight assemblages from LMP to Epi

Supplementary Fig. 17. Diachronic changes in mass of complete blanks in the eight assemblages from LMP to Epi

Supplementary Fig. 18. Diachronic changes in the use of stone tool raw material (chert) from MP to E Epi in southern Jordan

Supplementary Table 1. General composition of the stone-tool assemblages from Tor Sabiha, Tor Faraj, Wadi Aghar, Tor Fawaz, and Tor Hamar in southern Jordan

Supplementary Table 2. P-values of the pairwise comparisons in the edge length/mass ratios of bladelets by Dunn-Bonferroni test (two-sided)

Supplementary Table 3. P-values of the pairwise comparisons in the edge length/mass ratios of blades by Dunn-Bonferroni test (two-sided)

Supplementary Table 4. P-values of the pairwise comparisons in the edge length/mass ratios of flakes by Dunn-Bonferroni test (two-sided)

Supplementary References

Supplementary Note 1. Recent recognitions about the production rate of stone tool cutting-edges

Today, the validity of the classic model by Leroi-Gourhan[1] is known to be limited in light of later research progress in two major directions.

Firstly, the increase in Paleolithic archaeological records has revealed greater variability of lithic technologies and their space-time occurrences in more complicated patterns[2, 3, 4] rather than a simple unilinear change like that from Mode 1 to Mode 5[5]. As a result, a recent study of the cutting-edge production rates by Režek et al.[6] does not support a simple unilinear increase in the production rates over time but shows an increasing variability of the rates from the Lower Paleolithic to the Middle Paleolithic and then Upper Paleolithic.

Secondly, a series of experimental lithic production studies have clarified a wide range of flake products (i.e., debitage) that need to be considered for the accurate assessment of cutting-edge production rates[7, 8, 9, 10]. Some of these studies also examined how cutting-edge rates are correlated with quantitative morphological attributes of flakes, such as width, thickness, and the platform size, rather than typological categories of production methods (e.g., biface, Levallois, and blade)[8, 11]. This enabled more accurate assessment and deeper understanding of cutting-edge production rates and also revealed their variability. As a result, it is now recognized that the cutting-edge rates can vary considerably within the same general category of lithic production method, such as blade production, depending on more detailed technological choices, such as percussion techniques and platform preparations[8].

Supplementary Note 2. Background of the Initial Upper Paleolithic

The beginning of the Upper Palaeolithic in the Levant is characterized by several stone tool assemblages that have been called in various names, such as UP Phase 1[12], Emiran[13, 14, 15], the MP-UP transition[16], the Bokerian[17], and the Paléolithique intermédiaire[18]. Recently, these assemblages are often grouped under then term Initial Upper Paleolithic (hereafter, IUP)[19, 20]. The term IUP[21] was originally coined by referring to the lithic assemblage from the uppermost level (Level 4) at Boker Tachtit located in the southern Levantine region. Level 4 at Boker Tachtit is underlain by Levels 1–3 that yielded MP-UP transitional assemblages[22, 23, 24]. Other IUP sites in the Levant include Ksar Akil, Antelias, Abu Halka, Ücağızlı, Umm el-Tlel, Emireh, Tor Sadaf, Wadi Aghar, Tor Fawaz, Al-Ansab 2 among others[25].

IUP stone tool artifacts are generally characterized by both Middle Paleolithic and Upper Paleolithic techno-morphological features. Morphologically, retouched tools types in IUP assemblages include end scrapers and burins. These are the types that become prevalent in the Upper Paleolithic period. In addition, IUP assemblages in the Levant include unique types, such as Emireh points and chamfered pieces. Unmodified blanks in IUP assemblages characteristically include thick and wide blades that are often pointed. They usually have broad, often faceted, striking platforms. This platform type indicates the use of hard-hammer percussion for flaking rocks[22, 26, 27].

A few IUP sites in the Levant yielded human remains, but their taxonomic status has been controversial. At Üçağızlı, several human teeth were recovered from the IUP and Early Upper Paleolithic (Ahmarian) levels, and their morphologies have been reported to be "consistent with an attribution to *Homo sapiens*, but at least one possesses features more commonly associated with Neandertals"[26]. Modern human anatomy has also been suggested for a partial maxilla (Ethelruda) found in the IUP context (Level XXV) at Ksar Akil [28]. Another human fossil (Egbert) from Ksar Akil was discovered in the Early Upper Paleolithic (Ahmarian) deposits at Level XVII or XVIII and has been identified as a young individual of *Homo sapiens*[29].

The Levantine IUP lithic assemblages share basic techno-typological elements and chronological positions with those in Europe and Central–North Asia[30, 31, 32, 33, 34], and their occurrences were associated with the geographic expansion of *Homo sapiens* in those areas[30, 35]. The IUP technology is considered to have originated in the Levant on the basis of the old age of IUP assemblages at Boker Tachtit and the geographic position of the Levant bridging Africa and Eurasia.

Supplementary Discussion. Changes in cutting-edge production rates from the Late Middle Paleolithic to the Initial Upper Paleolithic

Our lithic assemblages from southern Jordan showed mixed results regarding the changes in cutting-edge production rates from LMP to IUP. While the Tor Faraj LMP assemblage showed a distinctively higher value in the total length/mass ratio than those of the IUP assemblages (Wadi Aghar and Tor Fawaz), Tor Sabiha LMP showed a rate only slightly higher than those of the IUP (Fig. 2). The slight difference between Tor Sabiha and the two IUP sites can be explained by the difference in the waste mass ratio between them. The lower ratio of waste mass in Tor Sabiha likely contributed to its slightly higher ratio of the total edge length to mass including wastes (Fig. 6).

Tor Faraj also differed from the IUP as it showed the significantly greater value in the length/mass ratio of individual pieces (Fig. 3a), which is more directly related to morphological variations in unmodified blanks. Specifically, unmodified blanks in the Tor Faraj assemblage were significantly thinner (and thus lighter) than those of the two IUP sites (Supplementary Figs. 15 and 17). Because the thickness of unmodified blanks and their mass show significant negative correlations with the edge length/mass ratio (Supplementary Fig. 9), thinner and lighter blanks at Tor Faraj must have contributed to its greater edge length/mass ratio than the IUP.

Lithic blank production at Tor Faraj was dominated by Levallois core reduction technology[36, 37, 38]. In contrast to a classic view[1], recent studies of lithic experimental production and mathematical modelling indicate that Levallois core technology (and similar discoidal core technology) can be productive in the number of blanks and the amount of cutting edge[7, 8, 39]. The productivity of Levallois core reduction can be particularly facilitated by recurrent methods that produce a wide range of blanks, in which differences between predetermined and predetermining blanks are gradual[40]. These recent views are consistent with the results of this study, which suggested specifically that the thickness of blanks is a key morphological attribute related to the cutting-edge productivity.

It is widely known that IUP blades are characterized by their robustness and large striking platforms that were probably implemented by hard-hammer percussion[22, 27, 41, 42]. These characteristics are usually highlighted in comparison with subsequent EUP slender blades/bladelets with small platforms, but this study suggests that the thickness and mass of IUP blanks are comparable to or greater than LMP blanks (Supplementary Figs. 15 and 17), and these morphological characteristics were major constraints in the cutting-edge productivity in the IUP.

Given the different results between Tor Faraj and Tor Sabiha, we need further analyses of other LMP assemblages to clarify variations in the cutting-edge productivity in the LMP. In any case, this study

at least suggests that the IUP blade production did not necessarily lead to an increase in the edge production rate from the LMP.

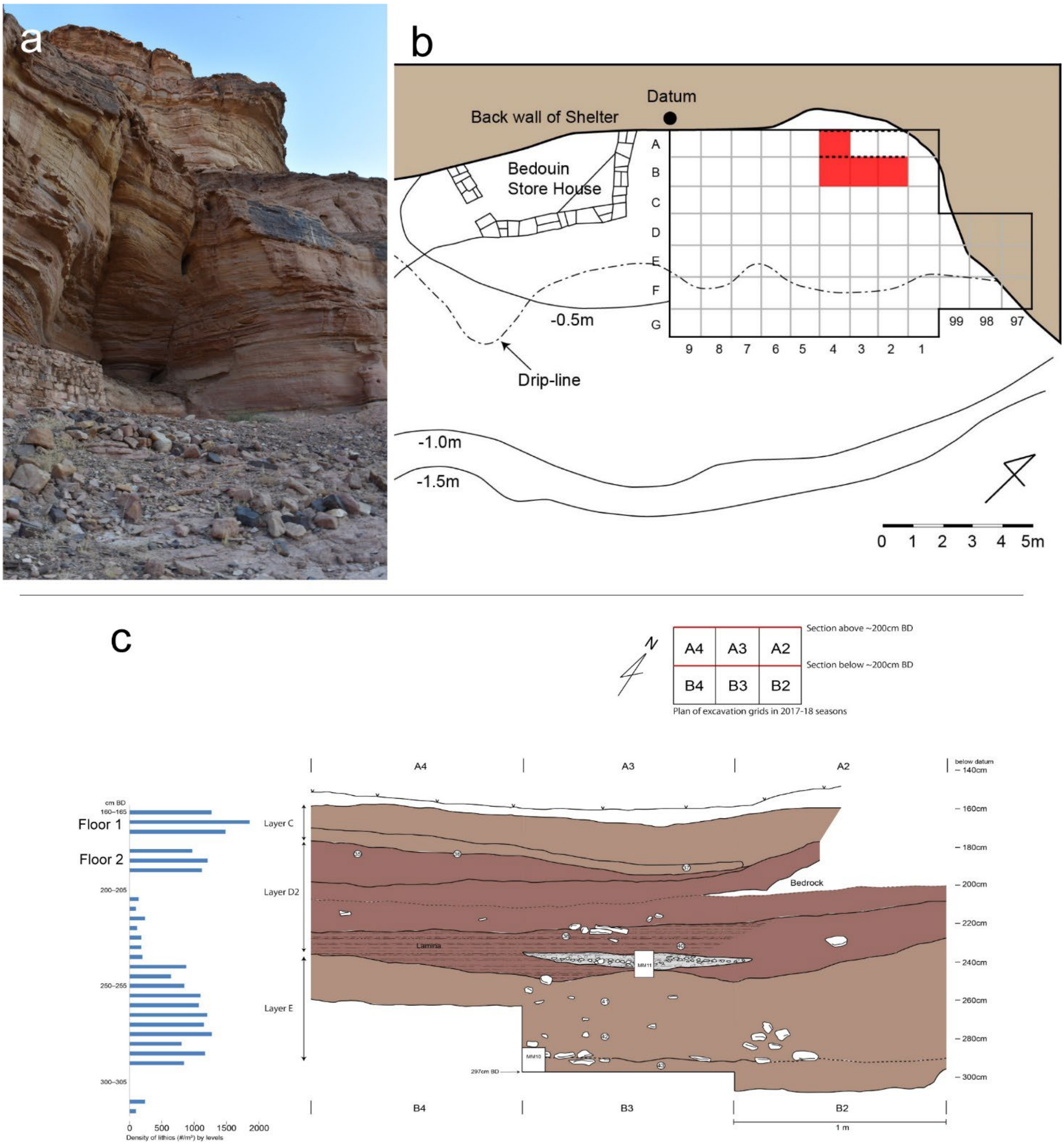

Supplementary Fig. 1. Overview, plan map, and stratigraphic section of Tor Faraj

(a) Overview of Tor Faraj. (b) Topographic map of Tor Faraj, showing the location of excavated areas (modified from ref.[38]. The re-excavation took place in Units A2, B2, B3, and B4 marked in red color. (c) Composite stratigraphic section (modified from ref.[43]). The upper part (above the dotted line near 200 cm BD) is the north wall of Units A2–A4 while the lower part (below the dotted line) is the north wall of Units B2–B4. A bar-graph shows lithic density (the number of lithics per 1 cm-thickness of deposits). Lithic artifacts analyzed in this study are from Layers D2 lower and E.

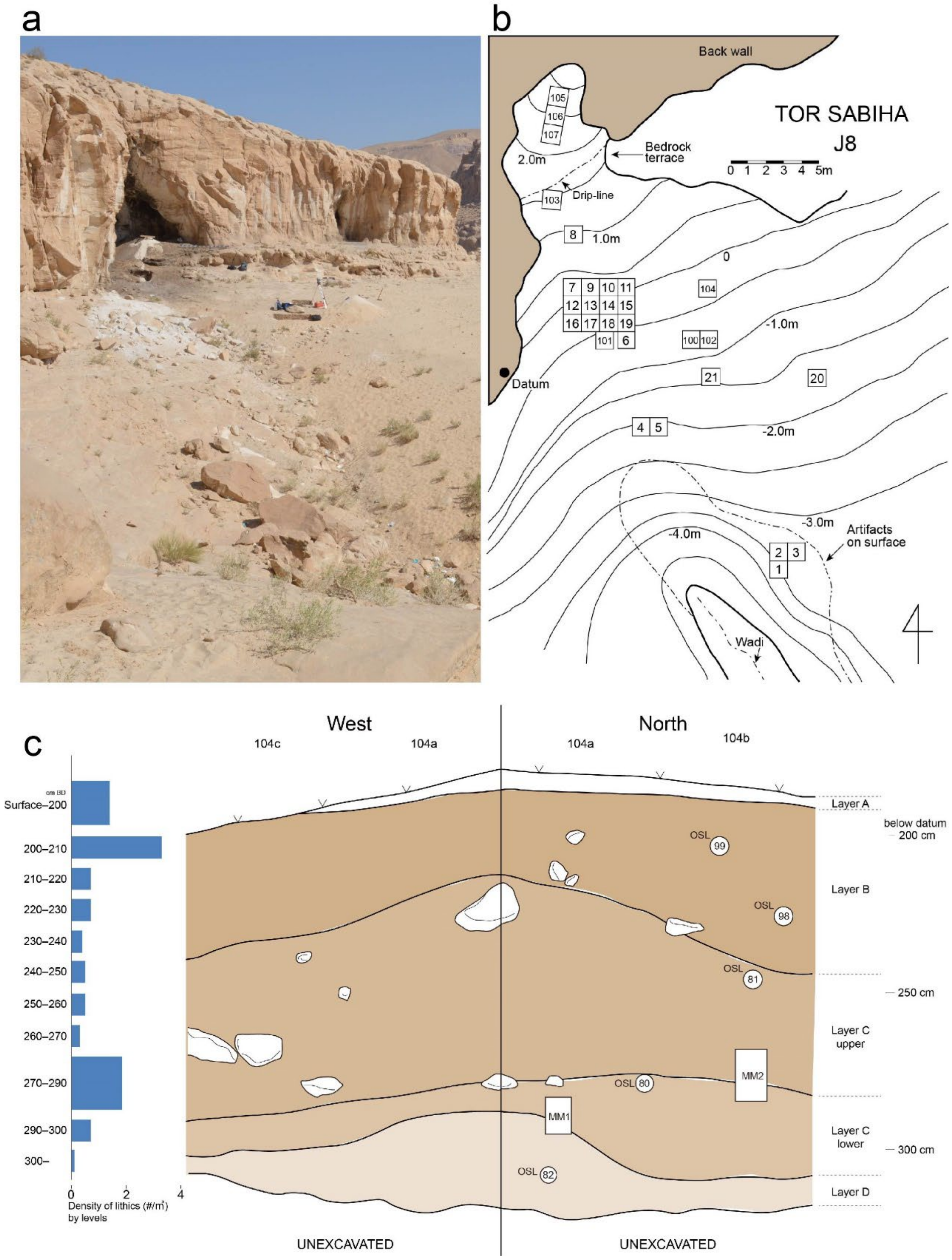

Supplementary Fig. 2. Overview, plan map, and stratigraphic section of Tor Sabiha

(a) Overview of Tor Sabiha. (b) Topographic map of Tor Sabiha, showing the location of excavated areas. Units 1–21 were excavated in 1979–1980[37] while Units 100–107 were excavated in 2019 and 2022. (c) Stratigraphic sections of Unit 104. A bar-graph shows lithic density (the number of lithics per 1 cm-thickness of deposits). Lithic artifacts analyzed in this study are from Layers C and D in Units 100, 102, and 104, and from Layer 11 in Units 105–107.

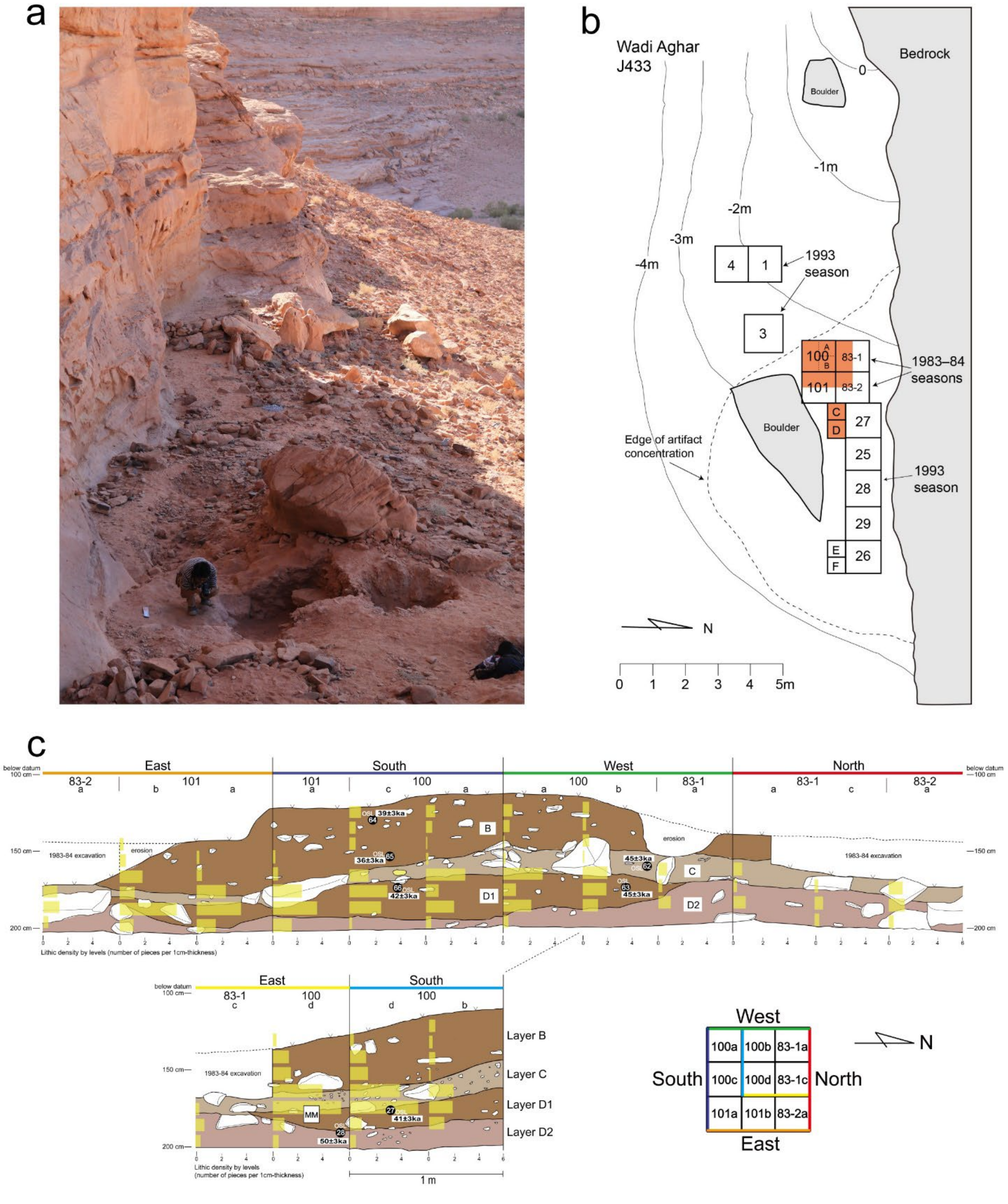

Supplementary Fig. 3. Overview, plan map, and stratigraphic section of Wadi Aghar

(a) Overview of an excavation area. (b) Topographic map showing the location of excavated areas. The renewed investigation since 2016 excavated Unit 100 and part of Units 101, 83-1, and 83-2 as well as Units C–F. Lithic artifacts analyzed in this study are from areas colored in red. (c) Stratigraphic sections showing the distribution of lithics and OSL dates. Bar-graphs show lithic density (the number of lithics per 1 cm-thickness of deposits by quadrats). Numbers in filled circles are the OSL sample # associated with dates (modified from ref.[44]).

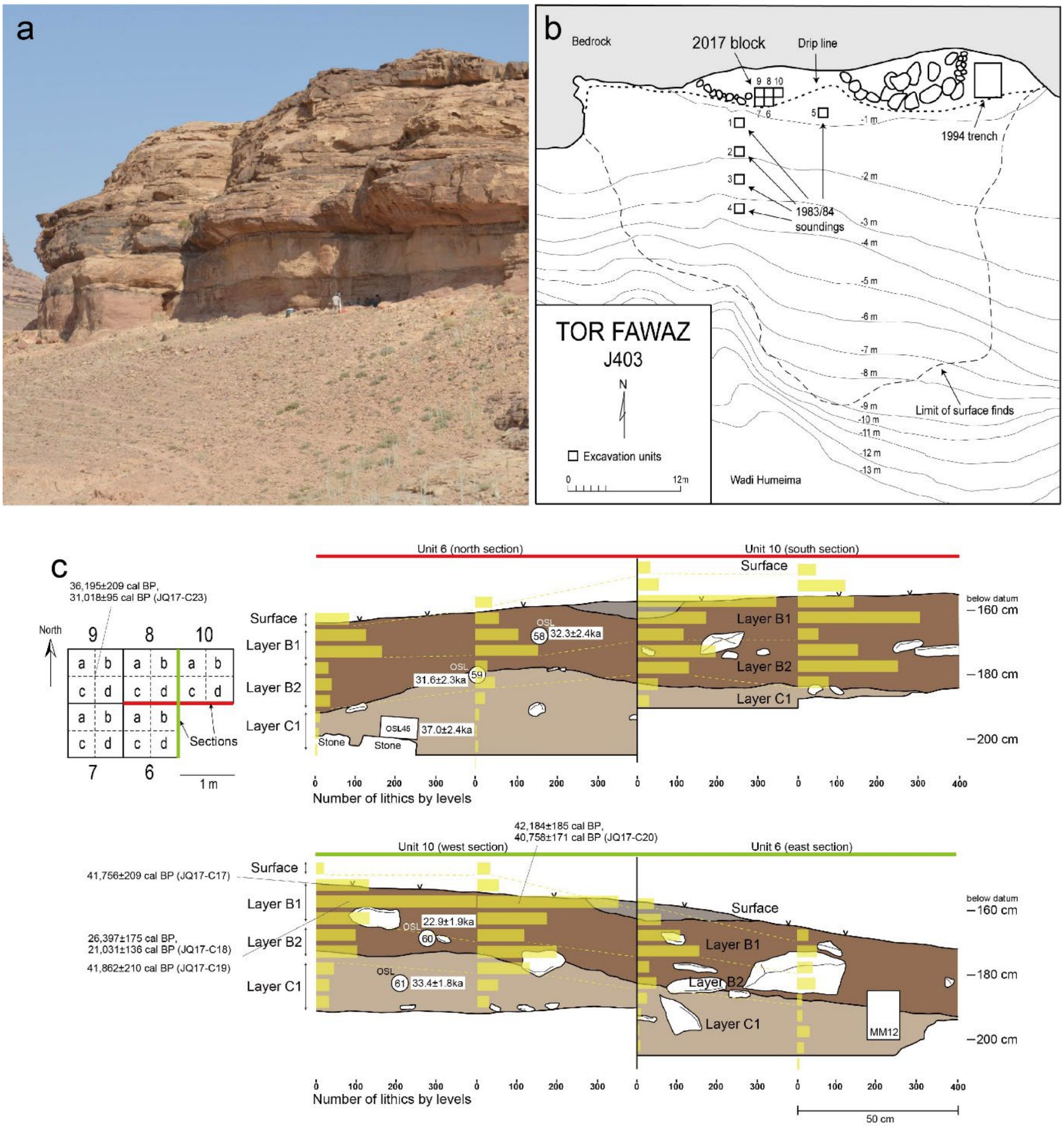

Supplementary Fig. 4. Overview, plan map, and stratigraphic section of Tor Fawaz

(a) Overview of Tor Fawaz. (b) Topographic map showing the location of excavated areas (modified from ref.[45]). Lithic artifacts analyzed in this study are from Units 6 and 10. (c) Stratigraphic section of Units 6 and 10, showing the vertical distribution of lithics. Bar graph shows the number of lithics by 5-cm-thick arbitrary levels. Numbers in circles are the OSL sample # associated with dates. The find spots of marine shells (JQ17-C17, C18, C19, C20, and C23) are indicated by their radiocarbon dates (See ref.[46] for more details).

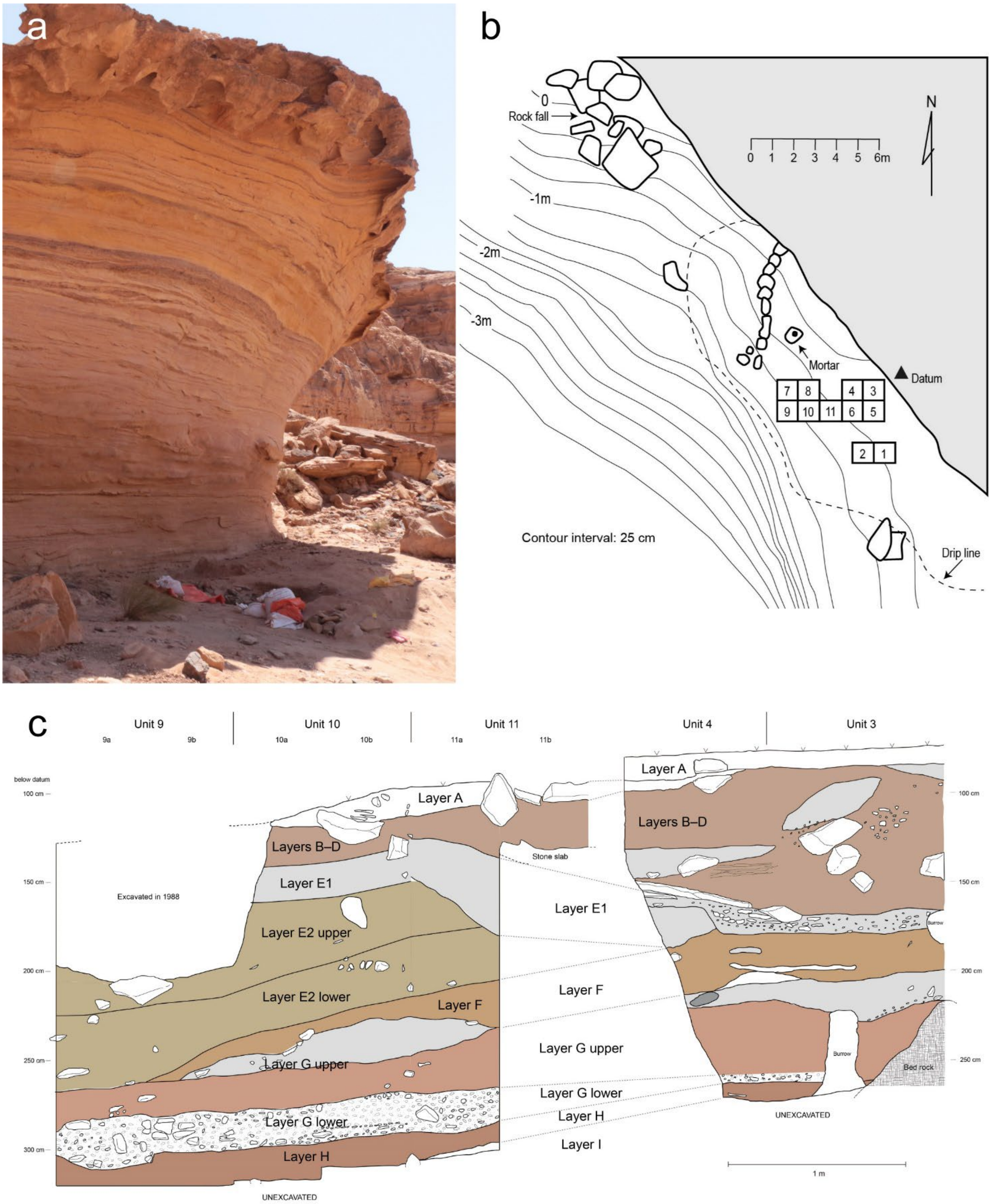

Supplementary Fig. 5. Overview, plan map, and stratigraphic section of Tor Hamar

(a) Overview of the excavation areas at Tor Hamar. (b) Topographic map showing the location of excavated squares (Units 1–11). (c) North stratigraphic sections of Units 3–4 and 9–11 at Tor Hamar (modified from ref.[47]). Layers A–E1 (Mushabian, Middle Epipaleolithic), Layer E2 (Qalkhan/Nebekian, Early Epipaleolithic), Layers F–H (Early Upper Paleolithic including Ahmarian).

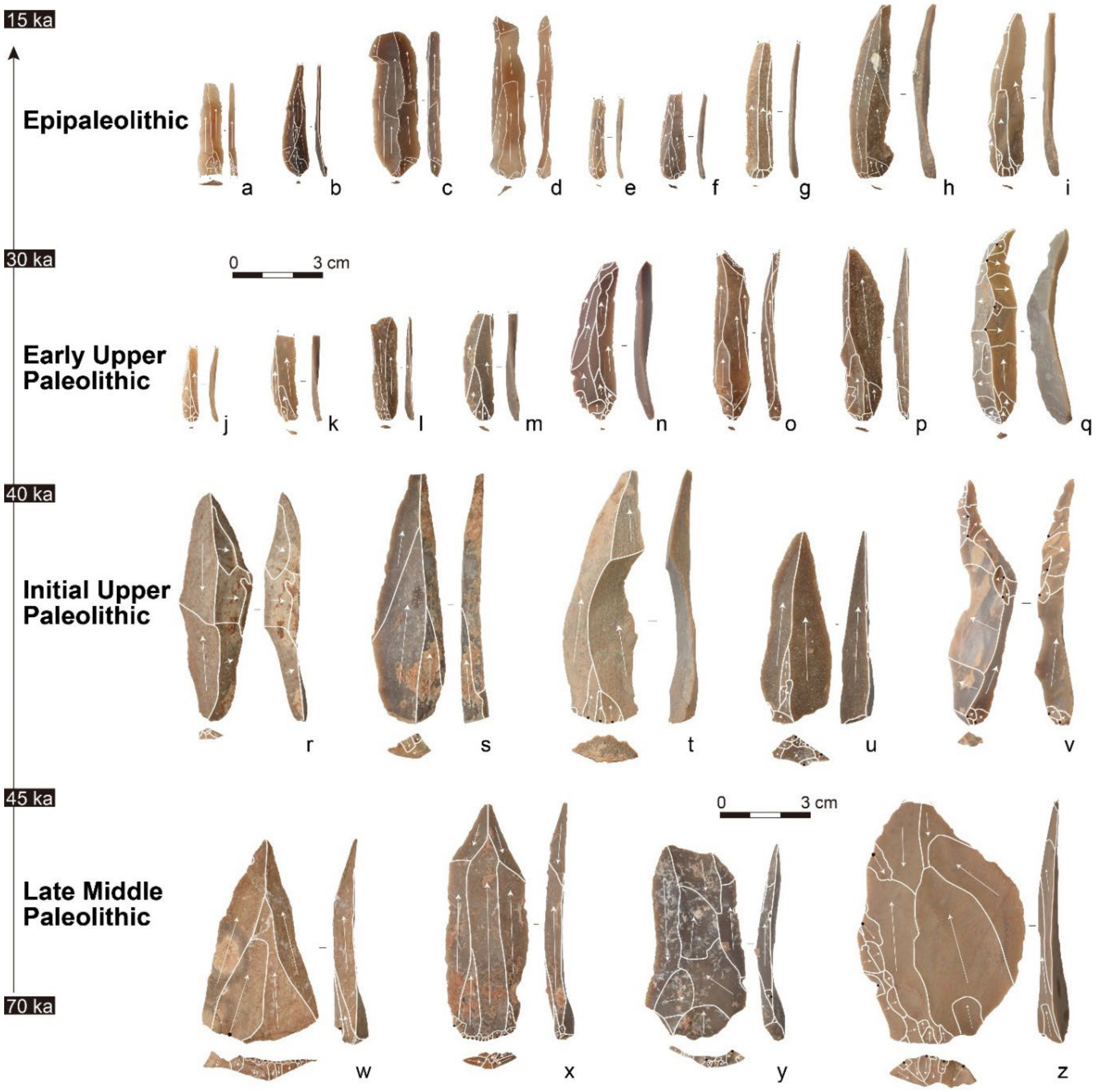

Supplementary Fig. 6. Some typical samples of flaked stone artifacts analyzed in this study

The stone artifacts are arranged in the chronological order from the bottom to top, marked with approximate dates (ka) and chrono-cultural names. (a–d) Middle Epipaleolithic (Mushabian) artifacts from Tor Hamar; (e–i) Early Epipaleolithic (Qalkhan/Nebekian) artifacts from Tor Hamar; (j–q) Early Upper Paleolithic (Ahmarian) artifacts from Tor Hamar; (r–v) Initial Upper Paleolithic artifacts from Wadi Aghar and Tor Fawaz; (w–z) Late Middle Paleolithic artifacts from Tor Faraj. (a, b, e–g, j–m) Bladelets; (c, d, h, i, n–p, r–u) blades; (q and v) core trimming elements, more specifically crested blades; (w) Levallois point; (x) Levallois blade; (y and z) Levallois flakes. Flaking scars are outlined, and arrows indicate flaking directions.

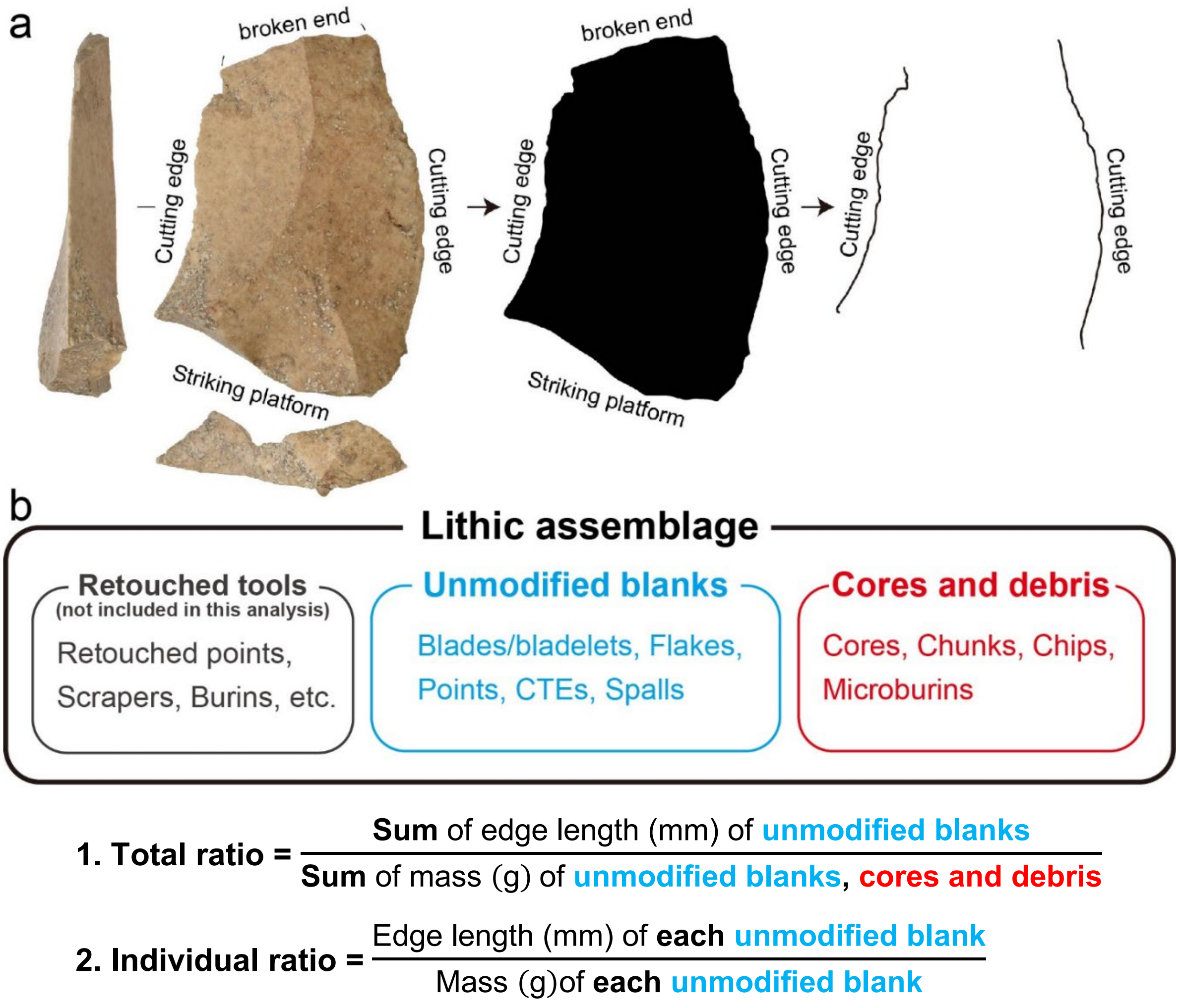

Supplementary Fig. 7. Summarized methods for quantifying the productivity of stone tool cutting-edges

(a) Three stages in the digital measurement of cutting-edge length. A photograph of an unmodified blank (left) is rendered to a polygon (middle), and then outlines of cutting-edges are extracted (right). Note that a striking platform and a broken end without sharp edges are excluded from the measurement to achieve a precise measurement of cutting-edge length. **(b)** Components of lithic assemblage, and their use in the calculations for two types of edge-length/mass ratios (mm/g). Each assemblage has a single value of the total ratio of edge length per mass while the individual ratios of edge-length/mass of unmodified blanks constitute a group of values for each assemblage.

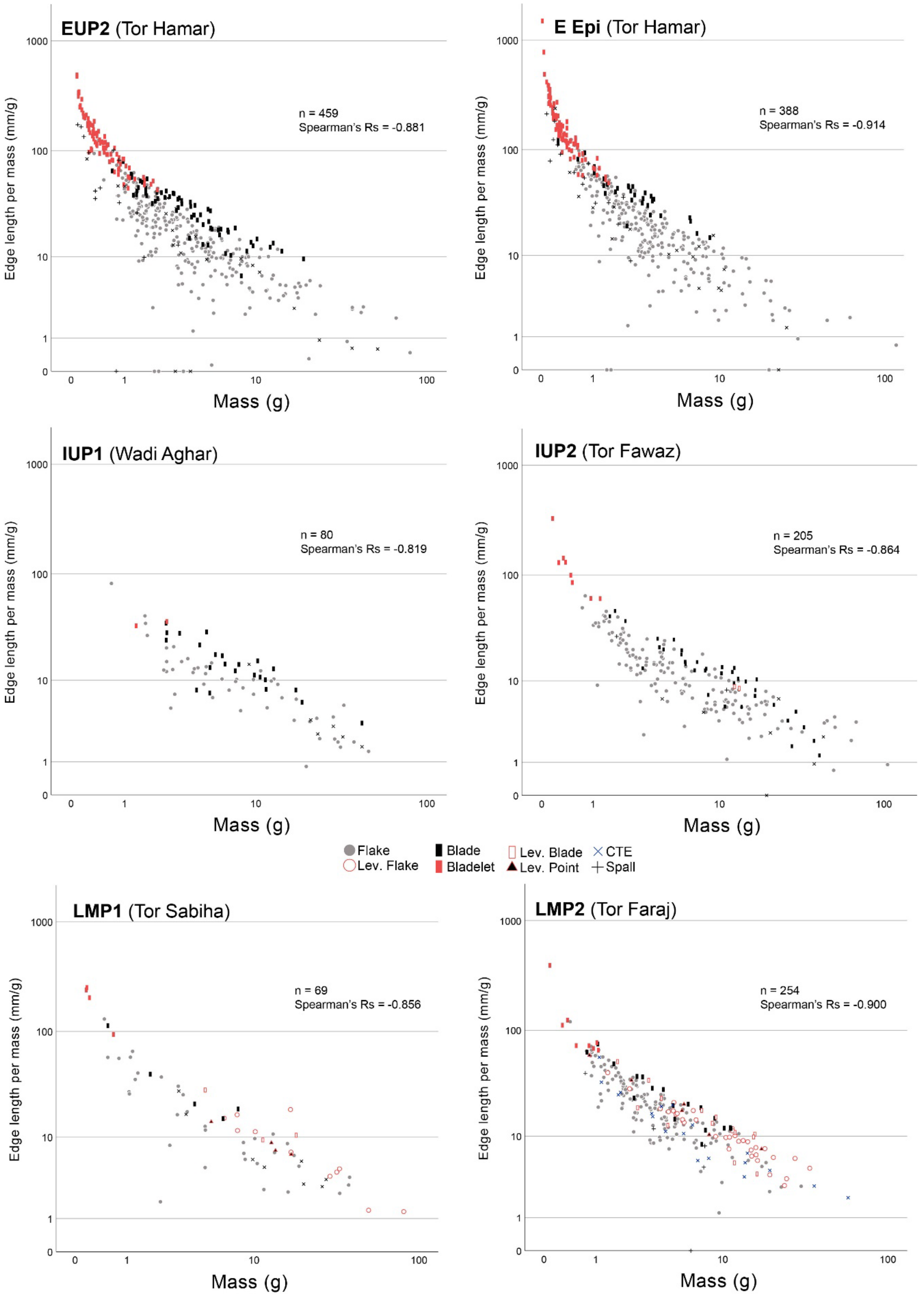

Supplementary Fig. 8. Scatterplots between the edge length per mass (mm/g) of complete lithic specimens and their mass

See Fig. 2 for the abbreviations of period-names. The plots include different symbols according to debitage types. Lev., Levallois; CTE, Core trimming element.

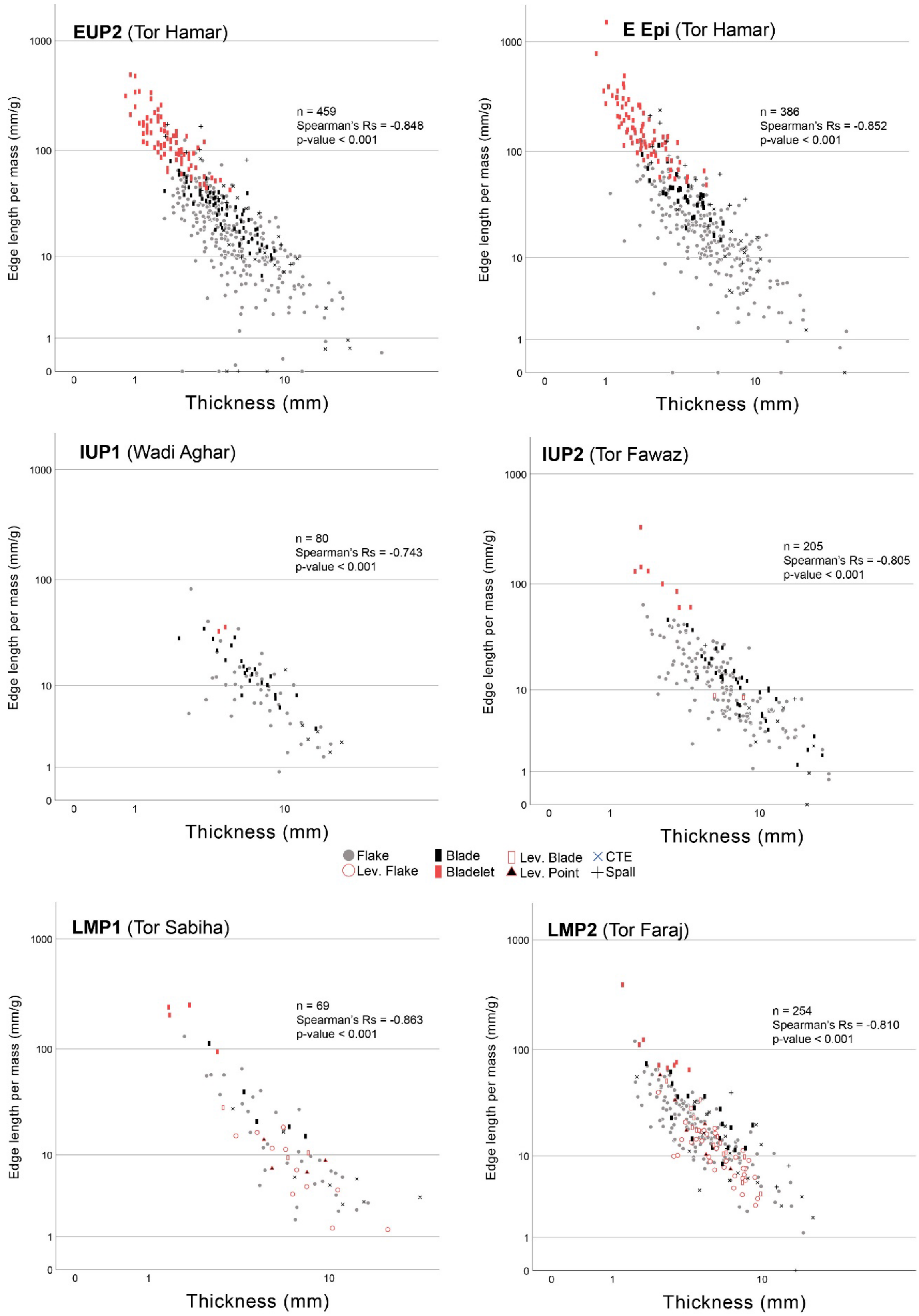

Supplementary Fig. 9. Scatterplots between the edge length per mass (mm/g) of complete lithic specimens and their thickness

See Fig. 2 for the abbreviations of period-names. The plots include different symbols according to debitage types. Lev., Levallois; CTE, Core trimming element. The significance testing of the Spearman correlation coefficient was two-sided.

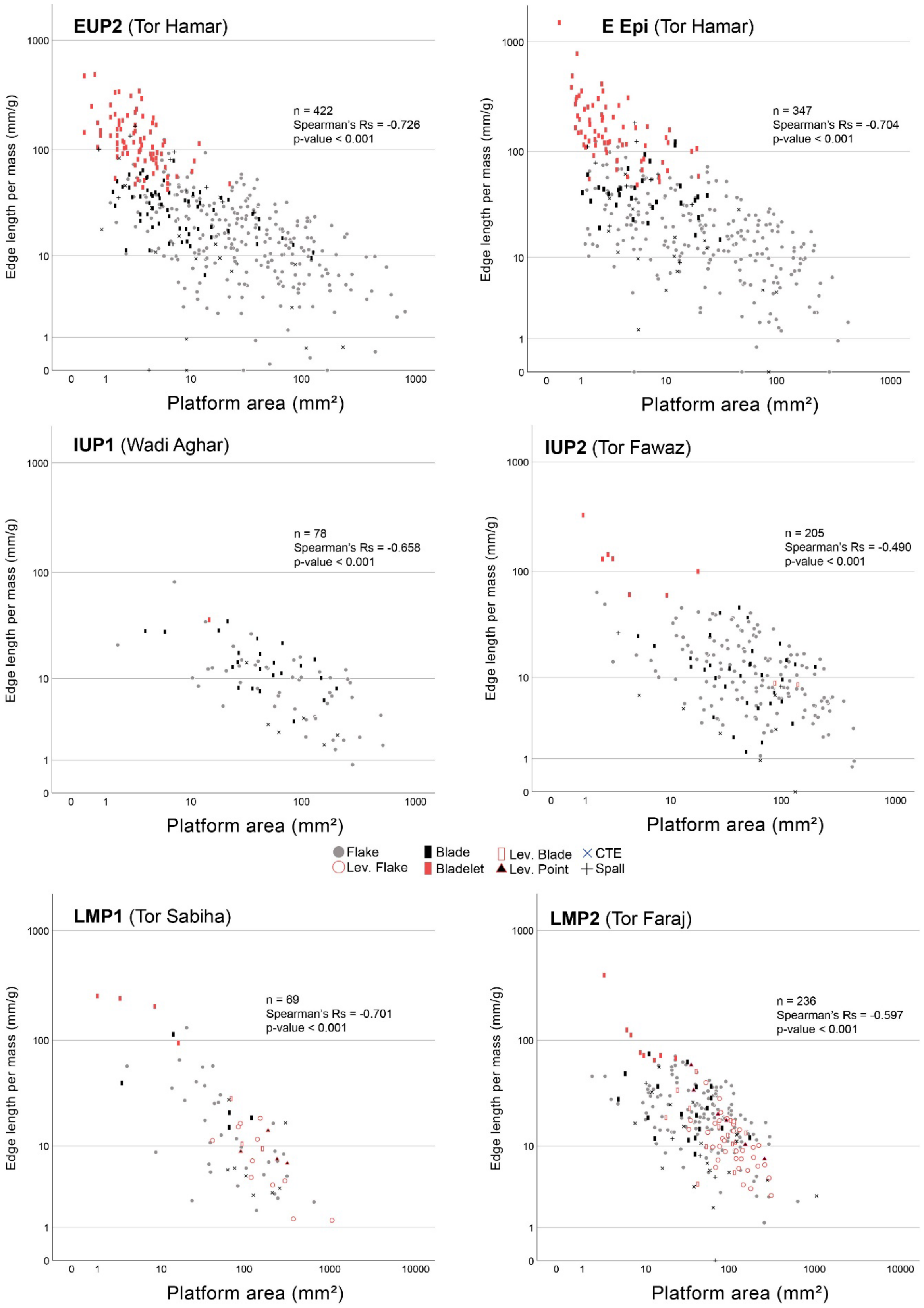

Supplementary Fig. 10. Scatterplots between the edge length per mass (mm/g) of complete lithic specimens and the platform area

See Fig. 2 for the abbreviations of period-names. The plots include different symbols according to debitage types. Lev., Levallois; CTE, Core trimming element. The significance testing of the Spearman correlation coefficient was two-sided.

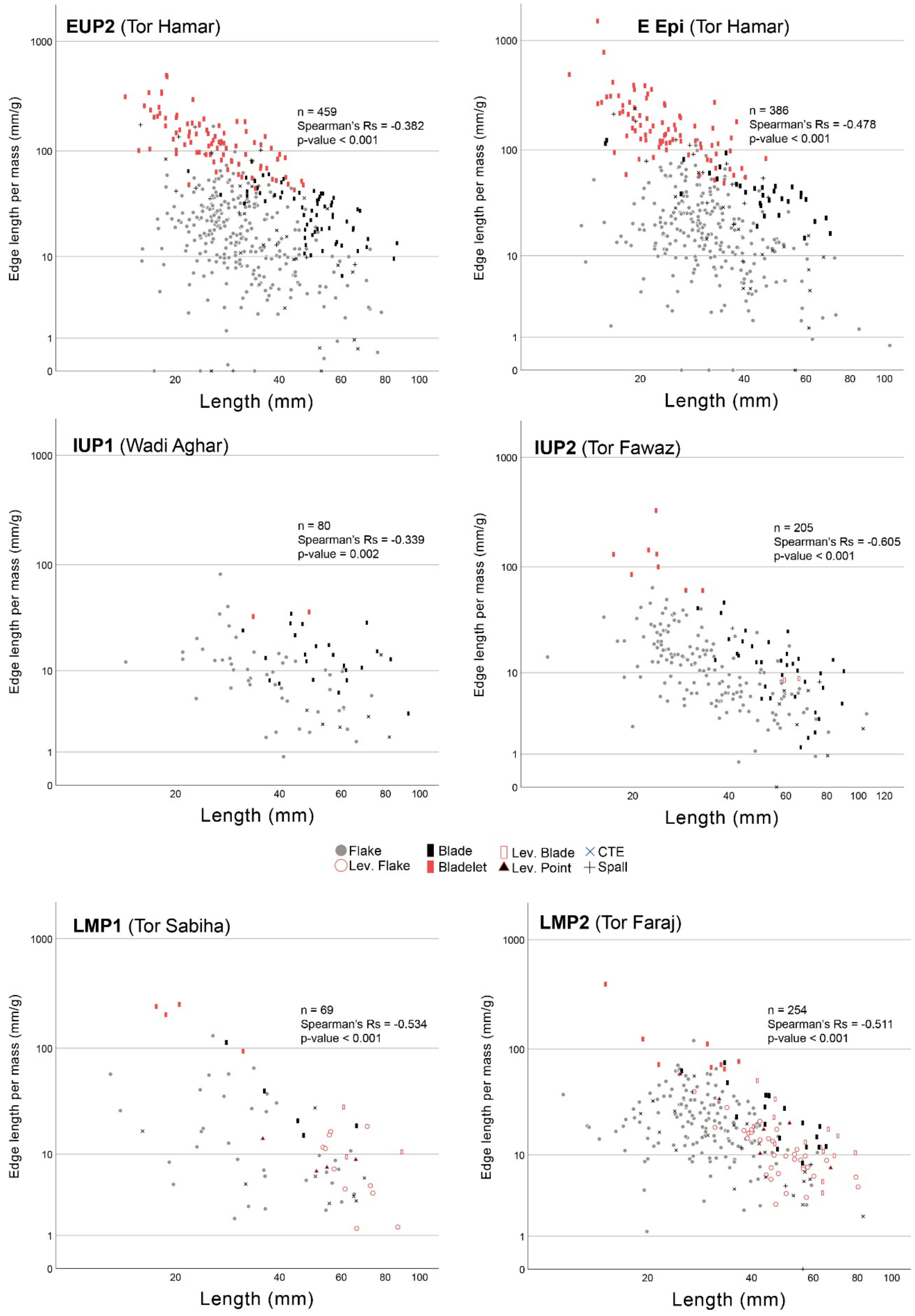

Supplementary Fig. 11. Scatterplots between the edge length per mass (mm/g) of complete lithic specimens and their length

See Fig. 2 for the abbreviations of period-names. The plots include different symbols according to debitage types. Lev., Levallois; CTE, Core trimming element. The significance testing of the Spearman correlation coefficient was two-sided.

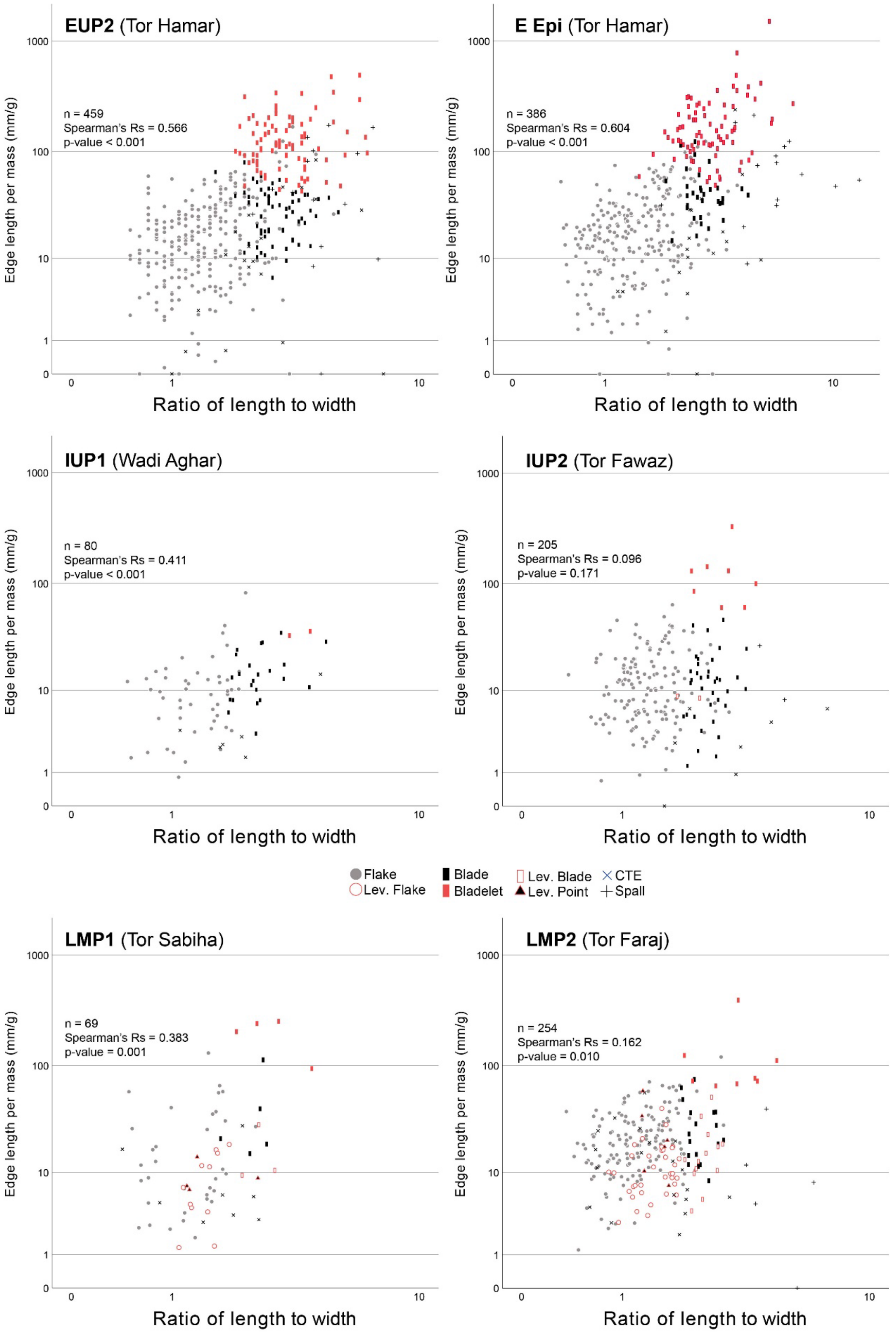

Supplementary Fig. 12. Scatterplots between the edge length per mass (mm/g) of complete lithic specimens and the ratio of length to width (i.e., elongatedness)

See Fig. 2 for the abbreviations of period-names. The plots include different symbols according to debitage types. Lev., Levallois; CTE, Core trimming element. The significance testing of the Spearman correlation coefficient was two-sided.

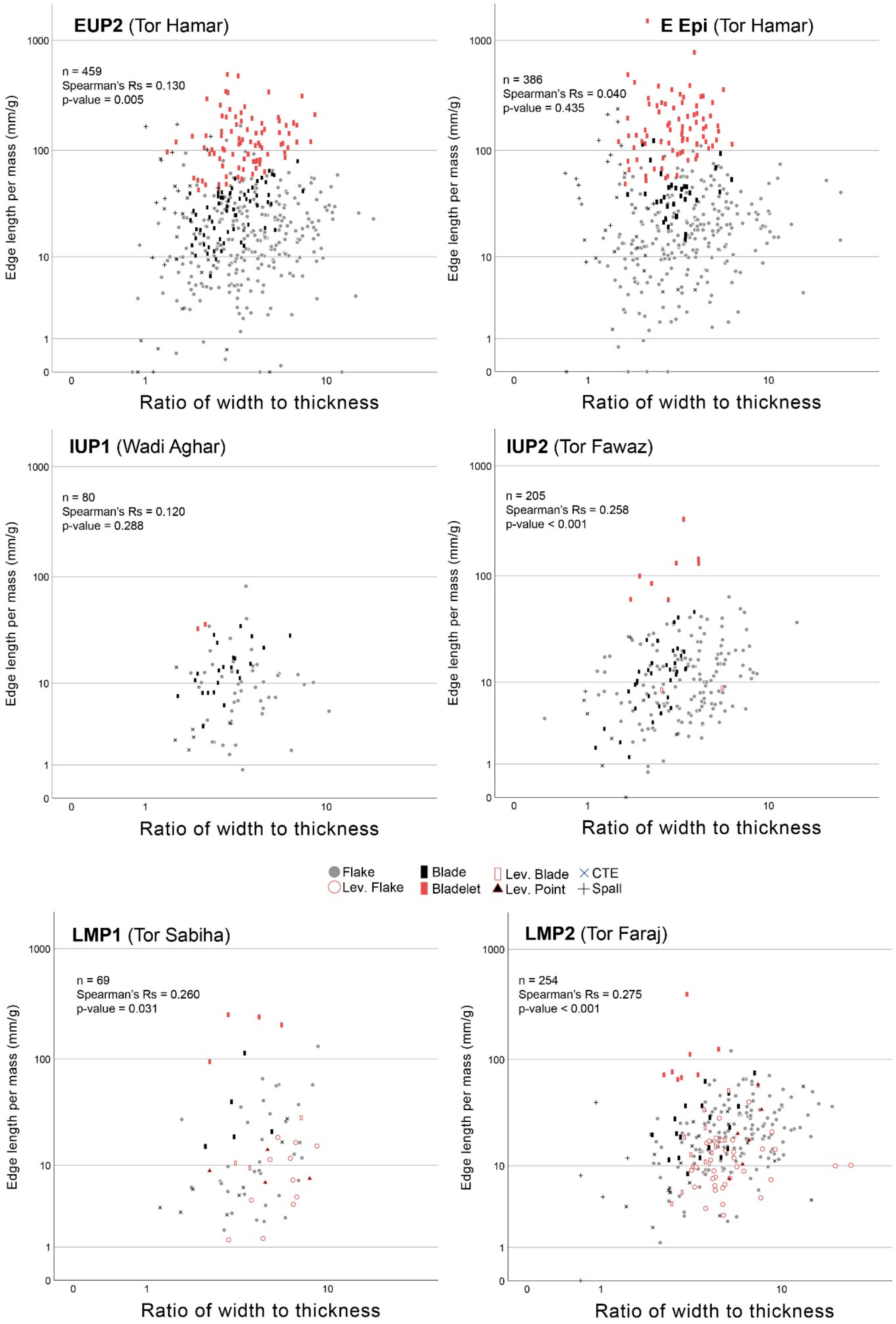

Supplementary Fig. 13. Scatterplots between the edge length per mass (mm/g) of complete lithic specimens and the ratio of width to thickness (i.e., flatness)

See Fig. 2 for the abbreviations of period-names. The plots include different symbols according to debitage types. Lev., Levallois; CTE, Core trimming element. The significance testing of the Spearman correlation coefficient was two-sided.

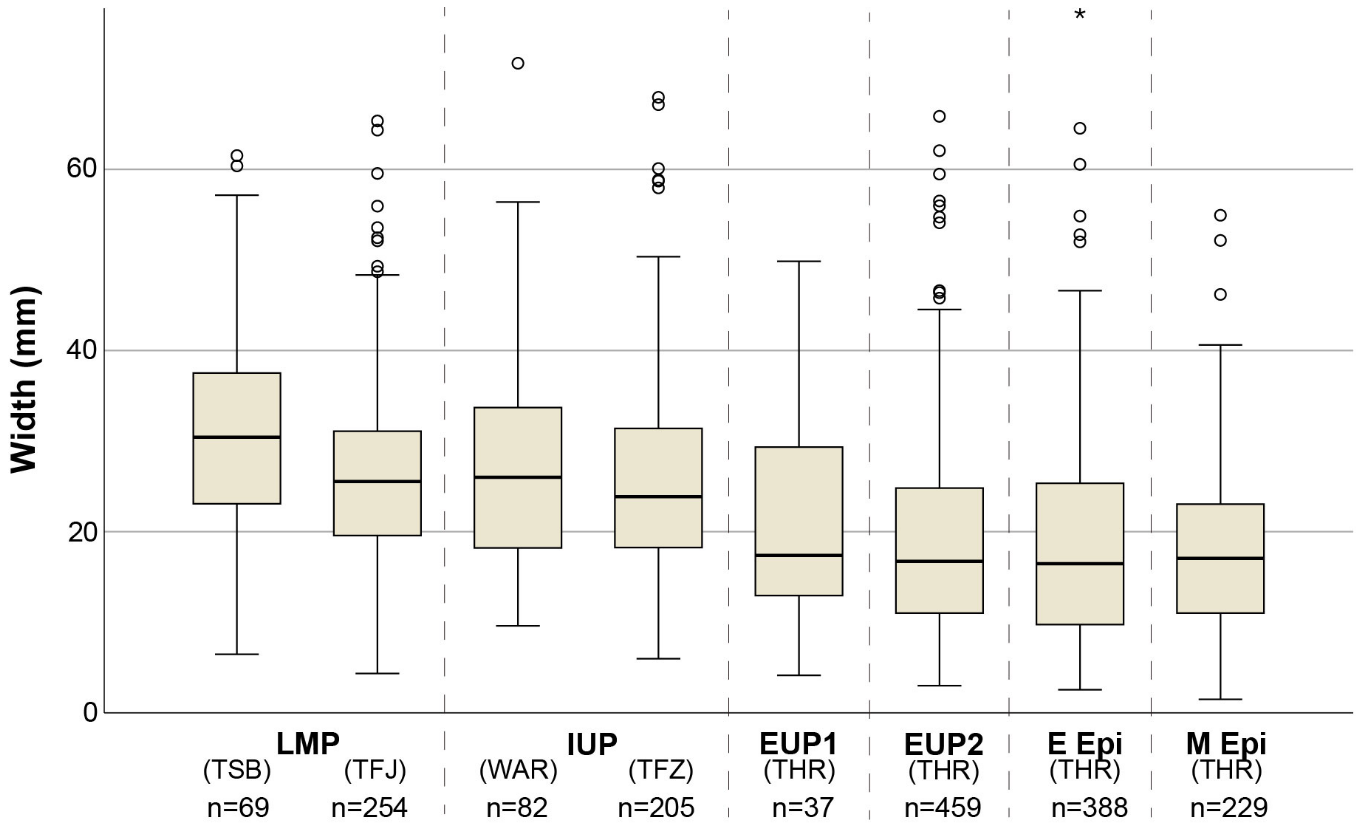

	LMP(TSB)	LMP(TFJ)	IUP(WAR)	IUP(TFZ)	EUP1	EUP2	E Epi	M Epi
LMP(TSB)		1.000	1.000	0.427	0.002	<0.001	<0.001	<0.001
LMP(TFJ)			1.000	1.000	0.037	<0.001	<0.001	<0.001
IUP(WAR)				1.000	0.055	<0.001	<0.001	<0.001
IUP(TFZ)					0.211	<0.001	<0.001	<0.001
EUP1						1.000	1.000	1.000
EUP2							1.000	1.000
E Epi								1.000
M Epi								

Supplementary Fig. 14. Diachronic changes in width of complete blanks in the eight assemblages from LMP to Epi

The box plots indicate median (middle line) and interquartile range (box). The upper and lower ends of whiskers represent maximum and minimum excluding outliers (single points) defined by the Tukey method. Note that we do not assume chronological relationship between LMP(TSB) and LMP(TFJ) and between IUP(WAR) and IUP(TFZ), as described in the text. The table shows p-values of the pairwise comparisons by Dunn-Bonferroni test (two-sided). The values lower than 0.05 are highlighted.

LMP, Late Middle Paleolithic; IUP, Initial Upper Paleolithic; EUP, Early Upper Paleolithic; E Epi, Early Epipaleolithic; M Epi, Middle Epipaleolithic. The lithic assemblages were excavated from Tor Sabiha (TSB), Tor Faraj (TFJ), Wadi Aghar (WAR), Tor Fawaz (TFZ), and Tor Hamar (THR) located in southern Jordan.

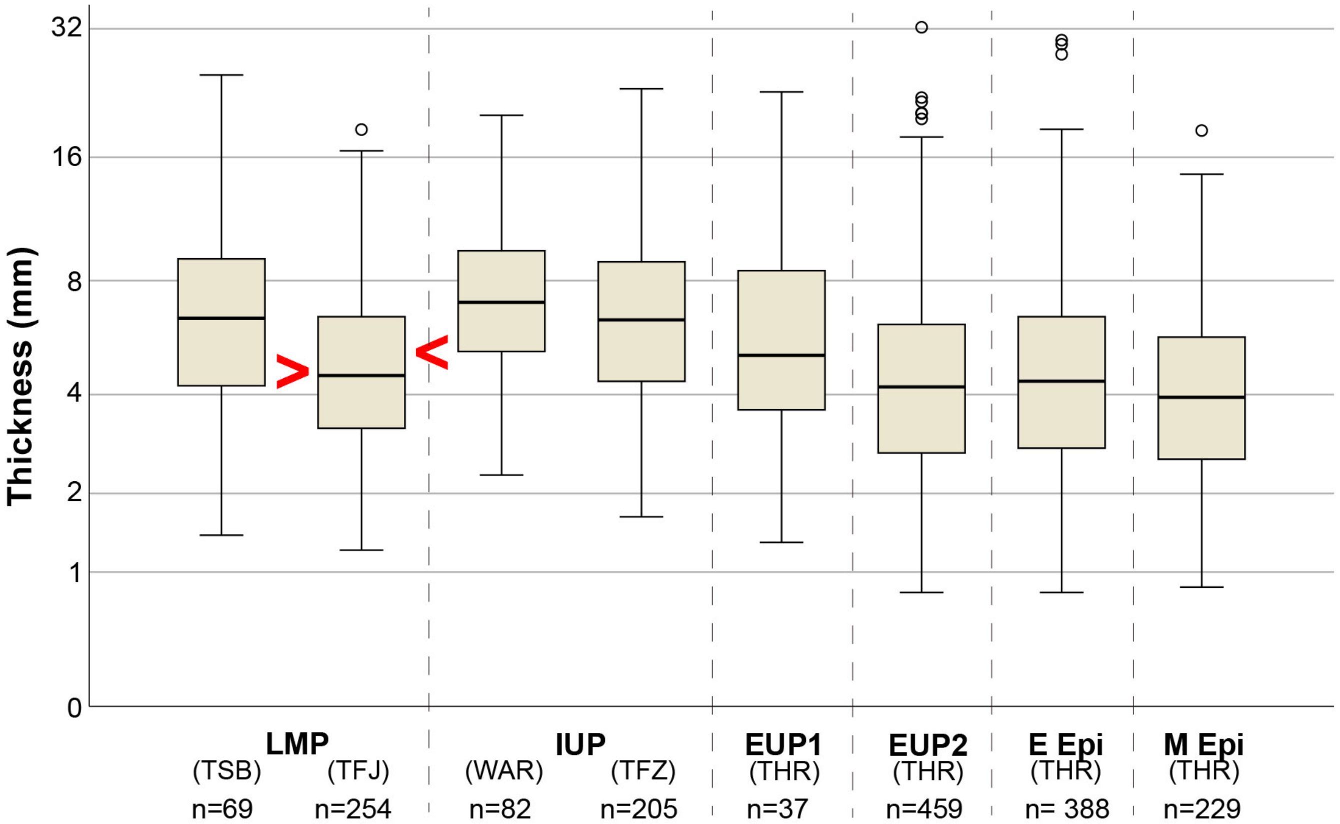

	LMP(TSB)	LMP(TFJ)	IUP(WAR)	IUP(TFZ)	EUP1	EUP2	E Epi	M Epi
LMP(TSB)		0.017	1.000	1.000	1.000	<0.001	<0.001	<0.001
LMP(TFJ)			<0.001	<0.001	1.000	1.000	1.000	0.059
IUP(WAR)				1.000	0.114	<0.001	<0.001	<0.001
IUP(TFZ)					1.000	<0.001	<0.001	<0.001
EUP1						1.000	1.000	0.220
EUP2							1.000	1.000
E Epi								1.000
M Epi								

Supplementary Fig. 15. Diachronic changes in thickness of complete blanks in the eight assemblages from LMP to Epi

The box plots indicate median (middle line) and interquartile range (box). The upper and lower ends of whiskers represent maximum and minimum excluding outliers (single points) defined by the Tukey method. Note that we do not assume chronological relationship between LMP(TSB) and LMP(TFJ) and between IUP(WAR) and IUP(TFZ), as described in the text. The table shows p-values of the pairwise comparisons by Dunn-Bonferroni test (two-sided). The values lower than 0.05 are highlighted. Inequality symbols (> and <) in box plots note statistically significant differences only for box plots next to each other.

LMP, Late Middle Paleolithic; IUP, Initial Upper Paleolithic; EUP, Early Upper Paleolithic; E Epi, Early Epipaleolithic; M Epi, Middle Epipaleolithic. The lithic assemblages were excavated from Tor Sabiha (TSB), Tor Faraj (TFJ), Wadi Aghar (WAR), Tor Fawaz (TFZ), and Tor Hamar (THR) located in southern Jordan.

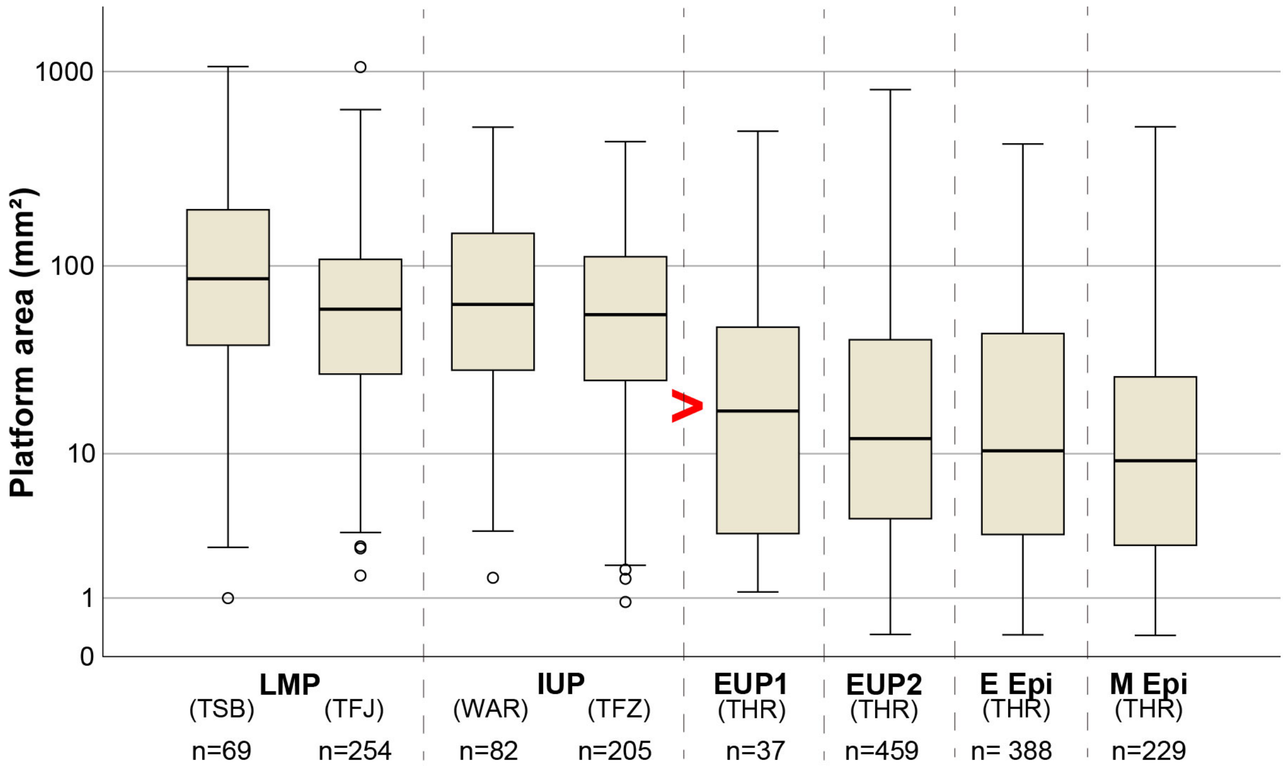

	LMP(TSB)	LMP(TFJ)	IUP(WAR)	IUP(TFZ)	EUP1	EUP2	E Epi	M Epi
LMP(TSB)		1.000	1.000	1.000	<0.001	<0.001	<0.001	<0.001
LMP(TFJ)			1.000	1.000	<0.001	<0.001	<0.001	<0.001
IUP(WAR)				1.000	<0.001	<0.001	<0.001	<0.001
IUP(TFZ)					0.001	<0.001	<0.001	<0.001
EUP1						1.000	1.000	1.000
EUP2							1.000	0.150
E Epi								1.000
M Epi								

Supplementary Fig. 16. Diachronic changes in the platform area of complete blanks in the eight assemblages from LMP to Epi

The box plots indicate median (middle line) and interquartile range (box). The upper and lower ends of whiskers represent maximum and minimum excluding outliers (single points) defined by the Tukey method. Note that we do not assume chronological relationship between LMP(TSB) and LMP(TFJ) and between IUP(WAR) and IUP(TFZ), as described in the text. The table shows p-values of the pairwise comparisons by Dunn-Bonferroni test (two-sided). The values lower than 0.05 are highlighted. Inequality symbols (> and <) in box plots note statistically significant differences only for box plots next to each other.

LMP, Late Middle Paleolithic; IUP, Initial Upper Paleolithic; EUP, Early Upper Paleolithic; E Epi, Early Epipaleolithic; M Epi, Middle Epipaleolithic. The lithic assemblages were excavated from Tor Sabiha (TSB), Tor Faraj (TFJ), Wadi Aghar (WAR), Tor Fawaz (TFZ), and Tor Hamar (THR) located in southern Jordan.

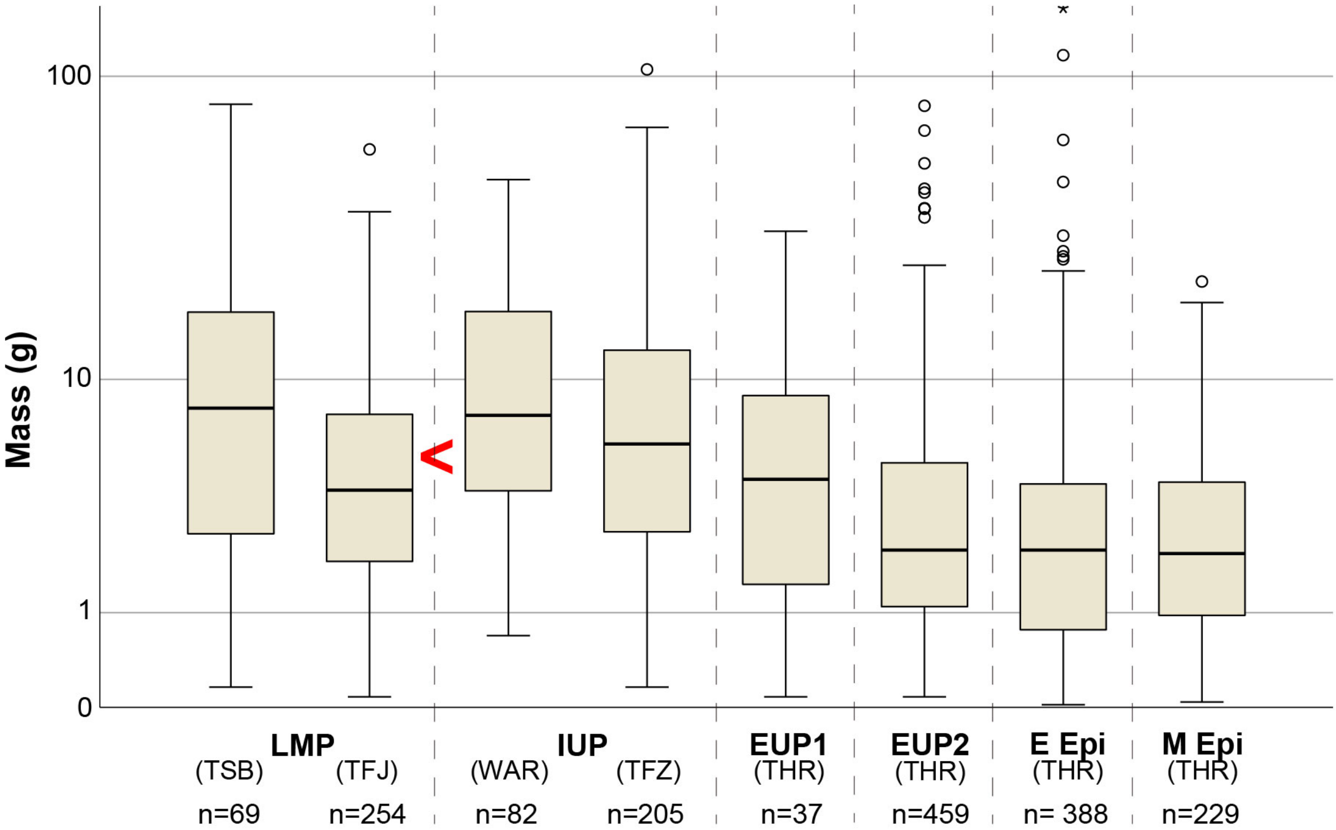

	LMP(TSB)	LMP(TFJ)	IUP(WAR)	IUP(TFZ)	EUP1	EUP2	E Epi	M Epi
LMP(TSB)		0.675	1.000	1.000	1.000	<0.001	<0.001	<0.001
LMP(TFJ)			0.001	0.018	1.000	<0.001	<0.001	<0.001
IUP(WAR)				1.000	0.035	<0.001	<0.001	<0.001
IUP(TFZ)					0.517	<0.001	<0.001	<0.001
EUP1						1.000	0.245	0.182
EUP2							1.000	1.000
E Epi								1.000
M Epi								

Supplementary Fig. 17. Diachronic changes in mass of complete blanks in the eight assemblages from LMP to Epi

The box plots indicate median (middle line) and interquartile range (box). The upper and lower ends of whiskers represent maximum and minimum excluding outliers (single points) defined by the Tukey method. Note that we do not assume chronological relationship between LMP(TSB) and LMP(TFJ) and between IUP(WAR) and IUP(TFZ), as described in the text. The table shows p-values of the pairwise comparisons by Dunn-Bonferroni test (two-sided). The values lower than 0.05 are highlighted. Inequality symbols (> and <) in box plots note statistically significant differences only for box plots next to each other.

LMP, Late Middle Paleolithic; IUP, Initial Upper Paleolithic; EUP, Early Upper Paleolithic; E Epi, Early Epipaleolithic; M Epi, Middle Epipaleolithic. The lithic assemblages were excavated from Tor Sabiha (TSB), Tor Faraj (TFJ), Wadi Aghar (WAR), Tor Fawaz (TFZ), and Tor Hamar (THR) located in southern Jordan.

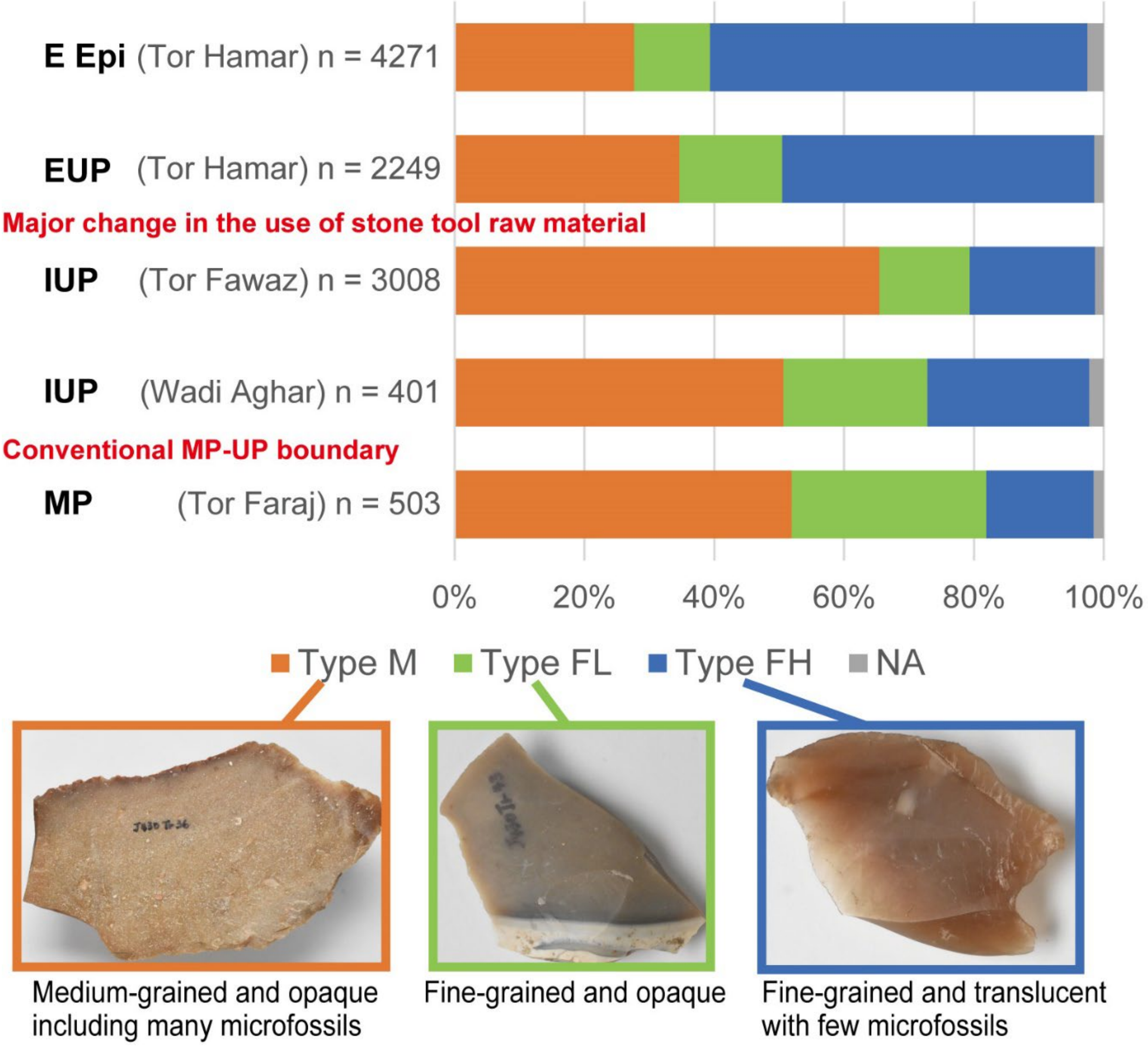

Supplementary Fig. 18. Diachronic changes in the use of stone tool raw material (chert) from MP to E Epi in southern Jordan

A major change in the use of stone raw material (i.e., increase in the use of Type FH chert) did not coincide with the conventional MP-UP boundary but occurred later in the EUP. See ref. [48] for the definition of chert types and data sources. Note that we do not assume chronological relationship between IUP (Wadi Aghar) and IUP (Tor Fawaz), as described in the text.

MP, Middle Paleolithic; IUP, Initial Upper Paleolithic; EUP, Early Upper Paleolithic; E Epi, Early Epipaleolithic.

Supplementary Table 1. General composition of the stone-tool assemblages from Tor Sabiha, Tor Faraj, Wadi Aghar, Tor Fawaz, and Tor Hamar in southern Jordan

The measurement of cutting-edge length was made excluding retouched tools, cores, chips and chunks. See Methods for the definitions of debitage types, such as blades/bladelets, core trimming elements, and chips. Dates (ka = kilo annum) are based on the results of several dating methods including radiocarbon (with calibration), optically stimulated luminescence, thermoluminescence, and amino-acid racemization (See Methods for details). Note that we do not assume chronological relationship between LMP (Tor Sabiha) and LMP (Tor Faraj) and between IUP (Wadi Aghar) and IUP (Tor Fawaz), as described in the text.

**Chrono-cultural unit**	Late Middle Paleolithic		Initial Upper Paleolithic		Early Upper Paleolithic (Ahmarian?)	Early Upper Paleolithic (Ahmarian)	Early Epipaleolithic (Qalkhan/ Nebekian)	Middle Epipaleolithic (Mushabian)
**Abbreviation**	LMP		IUP		EUP1	EUP2	E Epi	M Epi
**Site**	Tor Sabiha	Tor Faraj	Wadi Aghar	Tor Fawaz	Tor Hamar	Tor Hamar	Tor Hamar	Tor Hamar
**Dates**	69 ka	69–44 ka	45–40 ka	45–36 ka	Not dated	38–37 ka	24–18 ka	15.5–15.2 ka
**Excavation areas (Units)**	100–102, 104–107	A4, B2, B3, B4	100, 101, C, D, 83-1, 83-2	6a, 6b, 10a	9, 10, 11a, 11c	9, 10	9, 10a, 10c	11
**Layers**	Layers C and D of Units 100, 102, and 104. Layer 11 of Units 105–107	D2 lower, E	C, D1	Surface, B, C	H	F, G	E2	B–D, E1
**Retouched tools**	16	25	29	49	7	91	104	218
**Levallois points (unretouched)**	5	8	0	0	0	0	0	0
**Levallois blades**	6	15	0	2	0	0	0	0
**Levallois flakes**	13	40	0	0	0	0	0	0
**Blades**	4	44	57	173	17	175	122	149
**Bladelets**	8	22	14	57	34	407	418	239
**Partially cortical blades**	6	5	11	35	7	37	21	27
**Cortical/Partially cortical bladelets**	0	2	1	5	2	25	21	10
**Cortical blades**	1	2	2	8	0	7	0	2
**Flakes**	53	210	95	295	44	450	375	191
**Partially cortical flakes**	14	69	40	130	17	140	120	55
**Cortical flakes**	7	22	19	82	8	53	48	20
**Core trimming elements**	10	23	10	13	4	35	29	15
**Spalls**	0	5	1	5	2	33	25	5
**Microburin**	0	0	0	0	0	0	3	140
**Cores**	8	11	9	26	5	43	28	13
**Chips**	242	603	214	1130	219	2690	3054	2592
**Chunks**	0	3	3	6	1	16	19	6
**TOTAL**	393	1109	505	2016	367	4202	4387	3682
**References**	37	36, 37, 38, 43	36, 37, 44	36, 37, 45, 46	37, 43	36, 37, 43, 47	37, 43, 47	37, 43, 47

Supplementary Table 2. P-values of the pairwise comparisons in the edge length/mass ratios of bladelets by Dunn-Bonferroni test (two-sided)

The values lower than 0.05 are highlighted. Note that we do not assume chronological relationship between LMP(TSB) and LMP(TFJ) and between IUP(WAR) and IUP(TFZ), as described in the text.

	LMP(TSB)	LMP(TFJ)	IUP(WAR)	IUP(TFZ)	EUP1	EUP2	E Epi	M Epi
LMP(TSB)		1.000	0.073	0.450	1.000	1.000	1.000	1.000
LMP(TFJ)			0.203	1.000	1.000	1.000	0.517	1.000
IUP(WAR)				1.000	0.008	<0.001	<0.001	<0.001
IUP(TFZ)					0.023	<0.001	<0.001	<0.001
EUP1						1.000	1.000	1.000
EUP2							0.012	1.000
E Epi								1.000
M Epi								

Abbreviations: LMP = Late Middle Paleolithic; IUP = Initial Upper Paleolithic; EUP = Early Upper Paleolithic; E Epi = Early Epipaleolithic; M Epi = Middle Epipaleolithic. Site names: TSB = Tor Sabiha; TFJ = Tor Faraj; WAR = Wadi Aghar; TFZ = Tor Fawaz.

Supplementary Table 3. P-values of the pairwise comparisons in the edge length/mass ratios of blades by Dunn-Bonferroni test (two-sided)

The values lower than 0.05 are highlighted. Note that we do not assume chronological relationship between LMP(TSB) and LMP(TFJ) and between IUP(WAR) and IUP(TFZ), as described in the text.

	LMP(TSB)	LMP(TFJ)	IUP(WAR)	IUP(TFZ)	EUP1	EUP2	E Epi	M Epi
LMP(TSB)		1.000	0.231	0.721	1.000	1.000	1.000	1.000
LMP(TFJ)			0.050	0.250	1.000	0.407	<0.001	0.053
IUP(WAR)				1.000	0.009	<0.001	<0.001	<0.001
IUP(TFZ)					0.041	<0.001	<0.001	<0.001
EUP1						1.000	0.314	1.000
EUP2							0.002	1.000
E Epi								0.153
M Epi								

Abbreviations: LMP = Late Middle Paleolithic; IUP = Initial Upper Paleolithic; EUP = Early Upper Paleolithic; E Epi = Early Epipaleolithic; M Epi = Middle Epipaleolithic. Site names: TSB = Tor Sabiha; TFJ = Tor Faraj; WAR = Wadi Aghar; TFZ = Tor Fawaz.

Supplementary Table 4. P-values of the pairwise comparisons in the edge length/mass ratios of flakes by Dunn-Bonferroni test (two-sided)

The values lower than 0.05 are highlighted. Note that we do not assume chronological relationship between LMP(TSB) and LMP(TFJ) and between IUP(WAR) and IUP(TFZ), as described in the text.

	LMP(TSB)	LMP(TFJ)	IUP(WAR)	IUP(TFZ)	EUP1	EUP2	E Epi	M Epi
**LMP(TSB)**		1.000	0.078	0.029	1.000	1.000	1.000	0.812
**LMP(TFJ)**			<0.001	<0.001	0.037	1.000	1.000	1.000
**IUP(WAR)**				1.000	1.000	<0.001	<0.001	<0.001
**IUP(TFZ)**					1.000	<0.001	<0.001	<0.001
**EUP1**						0.282	0.161	0.008
**EUP2**							1.000	0.704
**E Epi**								1.000
**M Epi**								

Abbreviations: LMP = Late Middle Paleolithic; IUP = Initial Upper Paleolithic; EUP = Early Upper Paleolithic; E Epi = Early Epipaleolithic; M Epi = Middle Epipaleolithic. Site names: TSB = Tor Sabiha; TFJ = Tor Faraj; WAR = Wadi Aghar; TFZ = Tor Fawaz.

Supplementary References

1. Leroi-Gourhan A. *Gesture and Speech*. The MIT Press (1993).

2. Bar-Yosef O, Kuhn SL. The Big Deal about Blades: Laminar Technologies and Human Evolution. *American Anthropologist* **101**, 322–338 (1999).

3. Pargeter J, Shea JJ. Going big versus going small: Lithic miniaturization in hominin lithic technology. *Evol Anthropol* **28**, 72–85 (2019).

4. Nishiaki Y, Kadowaki S. Variability in lithic production technology during the range expansion of Paleolithic modern humans: Asian perspectives. *Quaternary International* **596**, 1–3 (2021).

5. Clark G. *World Prehistory: A New Synthesis*. Cambridge University Press (1969).

6. Režek Z, Dibble HL, McPherron SP, Braun DR, Lin SC. Two million years of flaking stone and the evolutionary efficiency of stone tool technology. *Nat Ecol Evol* **2**, 628–633 (2018).

7. Eren MI, Greenspan A, Sampson CG. Are Upper Paleolithic blade cores more productive than Middle Paleolithic discoidal cores? A replication experiment. *J Hum Evol* **55**, 952–961 (2008).

8. Muller A, Clarkson C. Identifying Major Transitions in the Evolution of Lithic Cutting Edge Production Rates. *PLoS One* **11**, e0167244 (2016).

9. Lycett SJ, Eren MI. Levallois economics: an examination of 'waste' production in experimentally produced Levallois reduction sequences. *Journal of Archaeological Science* **40**, 2384-2392 (2013).

10. Jennings TA, Pevny CD, Dickens WA. A biface and blade core efficiency experiment: implications for Early Paleoindian technological organization. *Journal of Archaeological Science* **37**, 2155–2164 (2010).

11. Lin SC, Rezek Z, Braun D, Dibble HL. On the Utility and Economization of Unretouched Flakes: The Effects of Exterior Platform Angle and Platform Depth. *American Antiquity* **78**, 724–745 (2013).

12. Neuville R. *Le Paléolithique et le Mésolithique du Désert de Judée*. Archives l'Institut de PaléontologieHumaine (1951).

13. Garrod DAE. A Transitional Industry from the Base of the Upper Palæolithic in Palestine and Syria. *The Journal of the Royal Anthropological Institute of Great Britain and Ireland* **81**, 121–130 (1951).

14. Shea J. *Stone tools in the Paleolithic and Neolithic Near East: a guide*. Cambridge University Press (2013).

15. Rose JI, Marks AE. "Out of Arabia" and the Middle-Upper Palaeolithic transition in the southern Levant. *Quartär* **61**, 49–85 (2014).

16. Marks AE. The Middle to Upper Paleolithic transition in the Levant. *Advances in world archaeology* **2**, 51–98 (1983).

17. Leder D. Lithic Variability and Techno-Economy of the Initial Upper Palaeolithic in the Levant. *International Journal of Archaeology* **6**, 23–36 (2018).

18. Boëda É, Bonilauri S, Kaltnecker E, Valladas H, Al-Sakhel H. Un débitage lamellaire au Proche-Orient vers 40 000 ans cal BP. Le site d'Umm el Tlel, Syrie centrale (A bladelet production to the Near-East about 40,000 (cal BP) years ago. The site of Umm el Tlel, central Syrie). *L'Anthropologie* **119**, 141–169 (2015).

19. Kuhn SL. In what sense is the Levantine Initial Upper Paleolithic a "transitional" industry? , 61–69 (2003).

20. Bar-Yosef O, Belfer-Cohen A. The Levantine Upper Palaeolithic and Epipalaeolithic. In: *South-eastern Mediterranean peoples between 130,000 and 10,000 years ago* (ed Garcea EAA). Oxbow Books (2010).

21. Marks AE, Ferring CR. The Early Upper Palaeolithic of the Levant. In: *The Early Upper Palaeolithic: evidence from Europe and the Near East* (eds Hoffecker JE, Wolf CA). B.A.R. (1988).

22. Marks A, Kaufman D. Boker Tachtit: the artifacts. In: *Prehistory and paleoenvironments in the central Negev, Israel: the Avdat/Aqev area, Part 3* (ed Marks A). Southern Methodist University (1983).

23. Boaretto E*, et al.* The absolute chronology of Boker Tachtit (Israel) and implications for the Middle to Upper Paleolithic transition in the Levant. *Proc Natl Acad Sci U S A* **118**, (2021).

24. Goder-Goldberger M, Barzilai O, Boaretto E. Innovative Technological Practices and their Role in the Emergence of Initial Upper Paleolithic Technologies: A View from Boker Tachtit. *Journal of Paleolithic Archaeology* **6**, (2023).

25. Goder-Goldberger M, Malinsky-Buller A. The Initial Upper Paleolithic and Its Place Within the Middle-to-Upper Paleolithic Transition of Southwest Asia: What Hides Behind the Curtain of Taxonomies? *Journal of Paleolithic Archaeology* **5**, (2022).

26. Kuhn SL*, et al.* The early Upper Paleolithic occupations at Ucagizli Cave (Hatay, Turkey). *J Hum Evol* **56**, 87–113 (2009).

27. Ohnuma K. *Ksar Akil Lebanon: A Technological Analysis of the Earlier Palaeolithic Levels of Ksar Akil, Volume III: Levels XXV-XIV*. B.A.R. (1988).

28. Douka K, Bergman CA, Hedges RE, Wesselingh FP, Higham TF. Chronology of Ksar Akil (Lebanon) and implications for the colonization of Europe by anatomically modern humans. *PLoS One* **8**, e72931 (2013).

29. Bergman CA, Stringer CB. Fifty years after: Egbert, an early Upper Palaeolithic juvenile from Ksar Akil, Lebanon. *Paléorient* **15**, 99–111 (1989).

30. Hublin JJ*, et al.* Initial Upper Palaeolithic Homo sapiens from Bacho Kiro Cave, Bulgaria. *Nature* **581**, 299–302 (2020).

31. Hublin J-J. The modern human colonization of western Eurasia: when and where? *Quaternary Science Reviews* **118**, 194–210 (2015).

32. Zwyns N. The Initial Upper Paleolithic in Central and East Asia: Blade Technology, Cultural Transmission, and Implications for Human Dispersals. *Journal of Paleolithic Archaeology* **4**, (2021).

33. Izuho M, Zwyns N, Kuhn S. Introduction of a Special Issue "Across steppes and mountains: the Initial Upper Paleolithic in Eurasia". *Journal of Paleolithic Archaeology* **4**, (2021).

34. Škrdla P. Comparison of Boker Tachtit and Stránská skála MP/UP Transitional Industries. *Journal of The Israel Prehistoric Society* **33**, 37–73 (2003).

35. Fu Q*, et al.* Genome sequence of a 45,000-year-old modern human from western Siberia. *Nature* **514**, 445–449 (2014).

36. Kadowaki S, Suga E, Henry DO. Frequency and production technology of bladelets in Late Middle Paleolithic, Initial Upper Paleolithic, and Early Upper Paleolithic (Ahmarian) assemblages in Jebel Qalkha, Southern Jordan. *Quaternary International* **596**, 4–21 (2021).

37. Henry DO. *Prehistoric cultural ecology and evolution*. Plenum Press (1995).

38. Henry DO. *Neanderthals in the Levant: Behavioral Organization and the Beginnings of Human Modernity*. Continuum (2003).

39. Brantingham PJ, Kuhn SL. Constraints on Levallois Core Technology: A Mathematical Model. *Journal of Archaeological Science* **28**, 747–761 (2001).

40. Shimelmitz R, Kuhn SL. The toolkit in the core: There is more to Levallois production than predetermination. *Quaternary International* **464**, 81–91 (2018).

41. Kuhn SL, Zwyns N. Rethinking the initial Upper Paleolithic. *Quaternary International* **347**, 29–38 (2014).

42. Azoury I. *Ksar Akil Lebanon: A Technological and Typological Analysis of the Transitional and Early Upper Palaeolithic Levels of Ksar Akil and Abu Halka, Volume I: Levels XXV - XII, Part ii Illustrations*. B.A.R. (1986).

43. Kadowaki S, Henry DO. Renewed investigation of the Middle and Upper Paleolithic sites in the Jebel Qalkha area, Southern Jordan. In: *Decades in Deserts: Essays on Near Eastern Archaeology in honour of Sumio Fujii* (eds S. Nakamura, T. Adachi, Abe M). Rokuichi Syobou (2019).

44. Kadowaki S*, et al.* Lithic technology, chronology, and marine shells from Wadi Aghar, southern Jordan, and Initial Upper Paleolithic behaviors in the southern inland Levant. *J Hum Evol* **135**, 102646 (2019).

45. Kerry KW, Henry DO. Tor Fawaz (J403): An Upper Palaeolithic Occupation in the Jebel Qalkha Area, Southwest Jordan. In: *More than Meets the Eye: Studies on Upper Palaeolithic Diversity in the Near East* (eds Goring-Morris AN, Belfer-Cohen A). Oxbow Books (2003).

46. Kadowaki S*, et al.* Lithic Technology and Chronology of Initial Upper Paleolithic Assemblages at Tor Fawaz, Southern Jordan. *Journal of Paleolithic Archaeology* **5**, (2022).

47. Naito YI*, et al.* Paleoenvironment and human hunting activity during MIS 2 in southern Jordan: Isotope records of prey remains and paleosols. *Quaternary Science Reviews* **282**, (2022).

48. Suga E, Ichinose N, Tsukada K, Kadowaki S, Massadeh S, Henry DO. Investigating changes in lithic raw material use from the Middle Paleolithic to the Upper Paleolithic in Jebel Qalkha, southern Jordan. *Archaeological Research in Asia* **29**, (2022).
