# Supplementary material for: Delayed increase in stone tool cutting-edge productivity at the Middle-Upper Paleolithic transition in southern Jordan
Source: Nat Commun. 2024 Feb 7;15:610. doi: 10.1038/s41467-024-44798-y (PMC10850154; doi:10.1038/s41467-024-44798-y)
Supplement: Supplementary file 3 — Reporting Summary [file 41467_2024_44798_MOESM3_ESM.pdf]

nature portfolio

Corresponding author(s): Seiji Kadowaki
Last updated by author(s): Dec 24, 2023

Reporting Summary

Nature Portfolio wishes to improve the reproducibility of the work that we publish. This form provides structure for consistency and transparency in reporting. For further information on Nature Portfolio policies, see our Editorial Policies and the Editorial Policy Checklist.

Statistics

For all statistical analyses, confirm that the following items are present in the figure legend, table legend, main text, or Methods section.

n/a	Confirmed	
☐	☒	The exact sample size (*n*) for each experimental group/condition, given as a discrete number and unit of measurement
☐	☒	A statement on whether measurements were taken from distinct samples or whether the same sample was measured repeatedly
☐	☒	The statistical test(s) used AND whether they are one- or two-sided<br>*Only common tests should be described solely by name; describe more complex techniques in the Methods section.*
☒	☐	A description of all covariates tested
☐	☒	A description of any assumptions or corrections, such as tests of normality and adjustment for multiple comparisons
☐	☒	A full description of the statistical parameters including central tendency (e.g. means) or other basic estimates (e.g. regression coefficient) AND variation (e.g. standard deviation) or associated estimates of uncertainty (e.g. confidence intervals)
☐	☒	For null hypothesis testing, the test statistic (e.g. *F*, *t*, *r*) with confidence intervals, effect sizes, degrees of freedom and *P* value noted<br>*Give P values as exact values whenever suitable.*
☒	☐	For Bayesian analysis, information on the choice of priors and Markov chain Monte Carlo settings
☒	☐	For hierarchical and complex designs, identification of the appropriate level for tests and full reporting of outcomes
☐	☒	Estimates of effect sizes (e.g. Cohen's *d*, Pearson's *r*), indicating how they were calculated

Our web collection on statistics for biologists contains articles on many of the points above.

Software and code

Policy information about availability of computer code

Data collection	We used Adobe Photoshop versions 17-23 and Adobe Illustrator versions 20-27 to measure the cutting-edge length of stone tools.
Data analysis	We used IBM SPSS version 27 and Microsoft Excel 2019 for the organization and analyses of the data.

For manuscripts utilizing custom algorithms or software that are central to the research but not yet described in published literature, software must be made available to editors and reviewers. We strongly encourage code deposition in a community repository (e.g. GitHub). See the Nature Portfolio guidelines for submitting code & software for further information.

Data

Policy information about availability of data

All manuscripts must include a data availability statement. This statement should provide the following information, where applicable:

- Accession codes, unique identifiers, or web links for publicly available datasets
- A description of any restrictions on data availability
- For clinical datasets or third party data, please ensure that the statement adheres to our policy

The data of cutting-edge length, mass, and several morphometric measurements of Paleolithic stone tools analyzed in this study are available to the public in the figshare repository. https://doi.org/10.6084/m9.figshare.23577093.

Research involving human participants, their data, or biological material

Policy information about studies with human participants or human data. See also policy information about sex, gender (identity/presentation), and sexual orientation and race, ethnicity and racism.

Reporting on sex and gender	The data used in this study were not generated from any experiment participated by humans or animals. Sex and gender are not considered in the design of this research.
Reporting on race, ethnicity, or other socially relevant groupings	This study does not use a framework related to race, ethnicity, or other socially constructed groupings.
Population characteristics	The data in this study were not generated from human research participants.
Recruitment	The data in this study were not generated from human research participants.
Ethics oversight	The data in this study were not generated from any experiment participated by humans or animals.

Note that full information on the approval of the study protocol must also be provided in the manuscript.

Field-specific reporting

Please select the one below that is the best fit for your research. If you are not sure, read the appropriate sections before making your selection.

☐ Life sciences ☐ Behavioural & social sciences ☒ Ecological, evolutionary & environmental sciences

For a reference copy of the document with all sections, see nature.com/documents/nr-reporting-summary-flat.pdf

Ecological, evolutionary & environmental sciences study design

All studies must disclose on these points even when the disclosure is negative.

Study description	We examined diachronic changes in the stone tool cutting-edge productivity during the Middle-Upper Paleolithic transition that critically coincided with wide dispersals of Homo sapiens in Eurasia. For this purpose, we used archaeological lithic specimens excavated from five sites in southern Jordan. The archaeological lithic materials were analyzed under a simple factorial design. Specifically, we examined two types of ratios of cutting-edge length to mass of stone tools as dependent variables, and we used the chrono-culture as the main factor, for which four main levels were set: Late Middle Paleolithic, Initial Upper Paleolithic, Early Upper Paleolithic, and Epipaleolithic. The latter two levels (i.e., Early Upper Paleolithic and Epipaleolithic) were further subdivided into two levels, thus making six levels in total. We also examined debitage types (e.g., blade, flake, and bladelet) as another factor. Additionally, we examined several dimensional attributes of lithics, such as length and width, as independent variables to see how they influence the cutting-edge length/mass ratios of lithics.
Research sample	We used eight lithic assemblages (n = 16,661 in total) that we excavated from five Paleolithic sites in southern Jordan. The assemblages represent a chrono-cultural sequence consisting of the Late Middle Paleolithic, the Initial Upper Paleolithic (IUP), the Early Upper Paleolithic (EUP), and the Epipaleolithic. The use of these materials is suitable for the purpose of this study because the sites are located close to each other (within 2 km) except for one site (Tor Sabiha), which is still only 14 km away and situated in the very similar environmental settings particularly in terms of the availability of lithic raw material, chert in this case. The study of these samples in the Levant, a key geographic area for the dispersals of Homo sapiens from Africa to Eurasia, can serve as a working hypothesis relevant to wide geographic regions including not only West Asia but also Europe and Central-North Asia, where similar IUP-EUP lithic technological changes have been observed.
Sampling strategy	All the lithic samples in this study were excavated by the team of the authors at five archaeological sites (Tor Sabiha, Tor Faraj, Wadi Aghar, Tor Fawaz, and Tor Hamar) in southern Jordan. The spatial and stratigraphic contexts of the samples have been recorded, and their chrono-cultural affiliations have been established in our previous studies, as described in the paper. The average number of edge-measured pieces in each lithic assemblage is 623 (min. = 127, max. = 1362), which is greater than the sample size (more or less 100) in previous studies that actually measured the cutting-edges of archaeological lithic artifacts. Even in experimental studies, it is rare to use more than 1000 lithic samples to estimate a cutting-edge production rate for each core-reduction technology, like Levallois, discoid, and prismatic blade. In addition, we tried to avoid retrieving excessive amounts of archaeological materials because the excavation inevitably causes disturbance of archaeological sites (i.e., removal of deposits and cultural remains).
Data collection	To collect data of the cutting-edge length of stone tools, we employed a digital method proposed by recent lithic experimental studies. As described in these studies, digital photos of lithic artifacts were processed in Adobe Photoshop to adjust the scale and to clarify the edges, which were then automatically traced by Adobe Illustrator to extract outlines. The length of the outlines was measured in Adobe Illustrator. We made close observations of each stone tool to exclude dull edges, such as striking platform, broken edges, and obtuse angles, from the measurement. Retouched tools were excluded from the analysis because of difficulty in estimating the original length of their cutting edge. We also did not measure the length of cutting edge of chips. Cores and chunks do not have sharp cutting-edges. Thus, we measured the cutting-edge length of unmodified blanks, which consists of several technomorphological types, such as flakes, blades, bladelets, and core trimming elements. The measurements of cutting-edge length and other morphometric attributes (e.g., mass and width) were performed by some of the authors: Ayami Watanabe,

Masato Hirose, Eiki Suga, and Seiji Kadowaki.

Timing and spatial scale

The lithic samples used in this study were collected by our excavations at five archaeological sites in southern Jordan that took place mainly in August and September in 2016, 2017, 2018, 2019, and 2022. After each time of the fieldwork, we exported the lithic materials to Nagoya University Museum, Japan, where we could spend sufficient time with necessary facilities to accumulate the data of cutting-edge length and several morphometric measurements. In this way, the collection of lithic data used in this study started in September 2016 and ended in January 2023.

Data exclusions

No data were excluded from the analysis.

Reproducibility

The chrono-cultural positions of the lithic assemblages used in this study were originally proposed by previous investigations in the 1980s and 1990s, and they were verified by the authors' recent re-investigations (since 2016) with new dating techniques and additional stone tool samples, as we describe in the paper. The measurement of cutting-edge length was repeated by independent persons for more than 1000 specimens, and we confirmed that the data are reproducible with only marginal inter-observer variations that would not affect the results and discussion presented in this paper.

Randomization

The lithic samples analyzed in this study consist of eight groups (lithic assemblages) that represent a chrono-cultural sequence from the Middle to Upper Paleolithic and then the Epipaleolithic. The chrono-cultural positions of the lithic assemblages are based on lithic techno-morphological attributes, their stratigraphic positions, and radiometric dates.

Blinding

The persons who mainly measured the lithic cutting-edge length were blinded from any expectation about the results (e.g., temporal trends in the edge production rates).

Did the study involve field work? ☒ Yes ☐ No

Field work, collection and transport

Field conditions

The lithic artifacts analyzed in this study were excavated from five rockshelter sites (Tor Sabiha, Tor Faraj, Wadi Aghar, Tor Fawaz, and Tor Hamar) located in the western Hisma Basin, southern Jordan. The current climate of the area is hyperarid, receiving less than 50 mm of annual rainfall, and physiographically transitional between the Irano-Turanian steppe and the Saharo-Arabian desert zones. We conducted excavations at the sites mainly in August and September in 2016, 2017, 2018, 2019, and 2022. The use of these materials is particularly suitable for the purpose of this study because the sites are located close to each other (within 2 km) except for one site (Tor Sabiha), which is still only 14 km away and situated in the very similar environmental settings particularly in terms of the availability of lithic raw material, chert in this case.

Location

Tor Sabiha (29°57'46.36"N, 35°28'10.30"E, 1195 m asl), Tor Faraj (29°56'19.9"N, 35°19'33.6"E, 985 m asl), Wadi Aghar (29° 56'11.99"N, 35°19'53.53"E, 965 m asl), Tor Fawaz (29°56'49.44"N, 35°20'9.03" E, 980 m asl), Tor Hamar (29°56'17.34"N, 35° 19'8.90"E, 985 m asl)

Access & import/export

Our archaeological fieldwork in Jordan was conducted according to the “Regulations for Archaeological Projects in Jordan based on the provisions of the Jordanian Antiquities Law Number 21 for the year 1988 and its amendments”. The permissions to conduct the fieldwork and to export archaeological materials to Japan, where analyses were conducted, were obtained from the Department of Antiquities of Jordan.
The following is the list of permissions for our fieldwork and the export of materials to Japan.
Excavation Permits: No. 2016/51 (issued on 24 July 2016), No. 2017/44 (issued on 16 August, 2017), No. 2018/18 (issued on 29 May, 2018), No. 2019/49 (issued on 15 August, 2019), and No. 2022/43 (issued on 21 August, 2022).
Export Permits: No. 12/5/2899 (issued on 15 August 2016), No. 12/5/3414 (issued on 18 September, 2017), No. 12/5/2290 (issued on 21 June, 2018), No. 12/5/382 (issued on 16 September, 2019), No. 12/5/35/7 (issued on 13 September, 2022)

Disturbance

The excavation of archaeological sites inevitably causes the removal of archaeological deposits and cultural remains. We minimized this extent around 5 square meters at each site, which provided the sufficient sample size of lithics for this study, as we described in the Sampling strategy section.

Reporting for specific materials, systems and methods

We require information from authors about some types of materials, experimental systems and methods used in many studies. Here, indicate whether each material, system or method listed is relevant to your study. If you are not sure if a list item applies to your research, read the appropriate section before selecting a response.

Materials & experimental systems

n/a	Involved in the study
☒	☐ Antibodies
☒	☐ Eukaryotic cell lines
☐	☒ Palaeontology and archaeology
☒	☐ Animals and other organisms
☒	☐ Clinical data
☒	☐ Dual use research of concern
☒	☐ Plants

Methods

n/a	Involved in the study
☒	☐ ChIP-seq
☒	☐ Flow cytometry
☒	☐ MRI-based neuroimaging

Palaeontology and Archaeology

Specimen provenance

The lithic artifacts analyzed in this study were excavated from five rockshelter sites (Tor Sabiha, Tor Faraj, Wadi Aghar, Tor Fawaz, and Tor Hamar) located in the western Hisma Basin, southern Jordan. Our archaeological fieldwork in Jordan was conducted according to the "Regulations for Archaeological Projects in Jordan based on the provisions of the Jordanian Antiquities Law Number 21 for the year 1988 and its amendments". The permissions for the fieldwork and the export of archaeological materials to Japan, where analyses were conducted, were obtained from the Department of Antiquities of Jordan.
The following is the list of permissions for our fieldwork and the export of materials to Japan.
Excavation Permits: No. 2016/51 (issued on 24 July 2016), No. 2017/44 (issued on 16 August, 2017), No. 2018/18 (issued on 29 May, 2018), No. 2019/49 (issued on 15 August, 2019), and No. 2022/43 (issued on 21 August, 2022).
Export Permits: No. 12/5/2899 (issued on 15 August 2016), No. 12/5/3414 (issued on 18 September, 2017), No. 12/5/2290 (issued on 21 June, 2018), No. 12/5/382 (issued on 16 September, 2019), No. 12/5/35/7 (issued on 13 September, 2022)

Specimen deposition

The stone tool specimens analyzed in this study have been stored in Nagoya University Museum, Japan.

Dating methods

No new dates are provided in this manuscript. The chrono-cultural positions of the analyzed lithic assemblages are based on our previous studies cited in the paper.

☐ Tick this box to confirm that the raw and calibrated dates are available in the paper or in Supplementary Information.

Ethics oversight

The permissions to conduct the archaeological fieldwork and export the excavated materials to Japan, where analyses were conducted, were obtained from the Department of Antiquities of Jordan.

Note that full information on the approval of the study protocol must also be provided in the manuscript.
